# Defects in the Expression of Chloroplast Proteins Leads to H_2_O_2_ Accumulation and Activation of Cyclic Electron Flow around Photosystem I

**DOI:** 10.3389/fpls.2016.02073

**Published:** 2017-01-13

**Authors:** Deserah D. Strand, Aaron K. Livingston, Mio Satoh-Cruz, Tyson Koepke, Heather M. Enlow, Nicholas Fisher, John E. Froehlich, Jeffrey A. Cruz, Deepika Minhas, Kim K. Hixson, Kaori Kohzuma, Mary Lipton, Amit Dhingra, David M. Kramer

**Affiliations:** ^1^Department of Plant Biology, Michigan State UniversityEast Lansing, MI, USA; ^2^DOE-Plant Research Laboratory, Michigan State UniversityEast Lansing, MI, USA; ^3^Institute of Biological Chemistry, Washington State UniversityPullman, WA, USA; ^4^Department of Horticulture, Washington State UniversityPullman, WA, USA; ^5^Department of Biochemistry and Molecular Biology, Michigan State UniversityEast Lansing, MI, USA; ^6^Environmental Molecular Sciences Laboratory, Pacific Northwest National LaboratoryRichland, WA, USA

**Keywords:** photosynthesis, cyclic electron flow around photosystem I, hydrogen peroxide (H_2_O_2_), Arabidopsis, chloroplast translation

## Abstract

We describe a new member of the class of mutants in Arabidopsis exhibiting high rates of cyclic electron flow around photosystem I (CEF), a light-driven process that produces ATP but not NADPH. High cyclic electron flow 2 (*hcef2*) shows strongly increased CEF activity through the NADPH dehydrogenase complex (NDH), accompanied by increases in thylakoid proton motive force (*pmf*), activation of the photoprotective q_E_ response, and the accumulation of H_2_O_2_. Surprisingly, *hcef2* was mapped to a non-sense mutation in the TADA1 (tRNA adenosine deaminase arginine) locus, coding for a plastid targeted tRNA editing enzyme required for efficient codon recognition. Comparison of protein content from representative thylakoid complexes, the cytochrome *bf* complex, and the ATP synthase, suggests that inefficient translation of *hcef2* leads to compromised complex assembly or stability leading to alterations in stoichiometries of major thylakoid complexes as well as their constituent subunits. Altered subunit stoichiometries for photosystem I, ratios and properties of cytochrome *bf* hemes, and the decay kinetics of the flash-induced thylakoid electric field suggest that these defect lead to accumulation of H_2_O_2_ in *hcef2*, which we have previously shown leads to activation of NDH-related CEF. We observed similar increases in CEF, as well as increases in H_2_O_2_ accumulation, in other translation defective mutants. This suggests that loss of coordination in plastid protein levels lead to imbalances in photosynthetic energy balance that leads to an increase in CEF. These results taken together with a large body of previous observations, support a general model in which processes that lead to imbalances in chloroplast energetics result in the production of H_2_O_2_, which in turn activates CEF. This activation could be from either H_2_O_2_ acting as a redox signal, or by a secondary effect from H_2_O_2_ inducing a deficit in ATP.

## Introduction

This work arose out of our attempts to understand how chloroplasts balance their energy budgets to efficiently capture solar energy and provide plants with sufficient energy for growth and maintenance while avoiding self-destructive side reactions, which led us to find unexpected connections between the maintenance of photosynthesis, the regulation of rigid stoichiometries of protein complexes in the chloroplast and the production of the reactive oxygen species H_2_O_2_.

In oxygenic photosynthesis, light is harvested by two distinct photochemical reaction centers, photosystem II (PSII) and photosystem I (PSI) that stimulate electron transfer through series of redox carriers to store solar energy in forms to drive biochemical processes (Eberhard et al., 2008). When PSI and PSII are electronically connected in series, they drive linear electron flow (LEF), which results in the oxidation of water and the reduction of NADPH. The electron transfer reactions of LEF are coupled to the uptake of protons from the chloroplast stroma and their deposition into the lumen, establishing an electrochemical gradient of protons, or proton motive force (*pmf*). Protons are taken up during reduction of plastoquinone at the Q_B_ site of PSII and the Q_i_ site of the cytochrome *bf* complex (*bf*). Protons are released into the lumen during water oxidation at the oxygen-evolving complex (OEC) of PSII and during plastoquinol oxidation at the Q_o_ site of the cytochrome *bf* complex. The *pmf* generated in these electron and proton transfer reactions drives the synthesis of ATP at the chloroplast ATP synthase.

The *pmf* also regulates the light reactions of photosynthesis through its effects on lumen pH-dependent q_E_ component of non-photochemical quenching (NPQ; reviewed in Müller et al., 2001), and electron flow through the cytochrome *bf* complex (Hope et al., 1994; Takizawa et al., 2007). The *pmf*, in turn, is modulated in response to environmental and metabolic conditions (Kanazawa and Kramer, 2002; Avenson et al., 2005; Cruz et al., 2005; Kohzuma et al., 2009; Strand and Kramer, 2014), allowing for fine-tuning of regulation of the light reactions in response to changes in metabolic state. The *pmf* can be controlled by regulating both rate of proton influx into the lumen, through the light-driven electron transfer reactions, and the efflux of protons from the lumen via the chloroplast ATP synthase, with different consequences on the balance of energy storage in ATP and NADPH and thus also affect downstream metabolic processes (Kramer et al., 2004; Cruz et al., 2005; Kramer and Evans, 2011).

The deposition of protons into the lumen is dependent on the rate of LEF. In addition, cyclic electron flow around PSI (CEF) is thought to contribute to *pmf*, and thus potentially to the activation of photoprotective mechanisms, and to augment the production of ATP to balance the ATP/NADPH energy budget (reviewed in Kramer and Evans, 2011).

CEF is a light-driven energy storing process that involves PSI but not PSII (Bendall and Manasse, 1995). Electrons from PSI are transferred to plastoquinone (PQ) forming plastoquinol (PQH_2_), which is subsequently oxidized by *bf* and shuttled back to PSI by plastocyanin (PC). The translocation of protons from the chloroplast stroma to the lumen through the Q-cycle, catalyzed by the *bf* complex (Cape et al., 2006; Cramer et al., 2011), contributes to the formation of *pmf* and ATP synthesis, but without net reduction of NADPH. In this way, CEF has been implicated in balancing the chloroplast energy budget by augmenting ATP production, and is thus thought to be physiologically important under conditions of elevated ATP demands. For example, CEF is known to be induced under environmental stresses such as drought (Kohzuma et al., 2009; Huang et al., 2012), high light (Takahashi et al., 2009), and chilling (Huang et al., 2010) when ATP may be needed to repair cellular machinery, maintain ion homeostasis, transport proteins, etc. CEF is also thought to be supply ATP for CO_2_ concentrating mechanisms, including the C_4_ cycle in plants (Takabayashi et al., 2005) and the carbon concentrating mechanism (CCM) in green algae, and appears to be critical under anoxia (Alric, 2014) or when CO_2_ is limiting (Lucker and Kramer, 2013) in the green alga *Chlamydomonas reinhardtii*. It is also possible that CEF plays a regulatory role in photosynthesis by acidifying the thylakoid lumen and thus activating the photoprotective q_E_ response and slowing electron flow at the *bf* complex (Munekage et al., 2002; Takahashi et al., 2009). However, it is important to recognize that uncontrolled activation of CEF will also result in a change in the ATP/NADPH output stoichiometry, a situation that can lead to deleterious secondary effects. Thus, chloroplasts also have alternate mechanisms of regulating lumen acidification that do not result in alteration of ATP/NADPH, including modulation of ATP synthase rates, and these appear to play primary roles in regulating photoprotection (reviewed in Strand and Kramer, 2014).

Several alternative CEF pathways have been proposed, that involve different PQ reductases, including the antimycin A sensitive ferredoxin:quinone reductase (FQR) pathway (Tagawa et al., 1963; Bendall and Manasse, 1995; Munekage et al., 2002; DalCorso et al., 2008; Alric, 2014), the Q_i_ site of the *bf* complex (Zhang et al., 2001; Joliot and Joliot, 2006), and the ferredoxin dehydrogenase complex (NDH, also referred to as the NADPH:plastoquinone oxidoreductase complex, though its substrate is Fd; Burrows et al., 1998; Sazanov et al., 1998). It is likely that different CEF pathways are activated in different species, and/or under different conditions (Casano et al., 2001; Lascano et al., 2003; Havaux et al., 2005; Takabayashi et al., 2005; Kohzuma et al., 2009; Iwai et al., 2010; Lucker and Kramer, 2013; Takahashi et al., 2013; Strand et al., 2015, 2016). To make matters more complex, a range of regulatory signals have been proposed for CEF, including sensing of ATP/ADP ratios (Joliot and Joliot, 2002, 2006), chloroplast redox status (Breyton et al., 2006; Takahashi et al., 2013; Alric, 2014; Johnson et al., 2014), metabolic intermediates (Fan et al., 2007), state transitions (Finazzi et al., 2002; Iwai et al., 2010), calcium (Terashima et al., 2012), and reactive oxygen species (Casano et al., 2001; Lascano et al., 2003; Strand et al., 2015).

To address these questions, we initiated an effort to discover new CEF structural and regulatory components by isolating mutants of Arabidopsis (*Arabidopsis thaliana*) with constitutively elevated CEF, which we named high cyclic electron flow (*hcef*) mutants (Livingston et al., 2010a,b). The first of these mutants to be reported, *hcef1*, was mapped to a missense mutation in the chloroplast-targeted fructose 1,6 bisphosphatase (FBPase), and appears to indirectly activate CEF by disrupting redox balance (Livingston et al., 2010b; Strand et al., 2015) possibly by activating a futile metabolic cycle that consumes ATP (Livingston et al., 2010a; Sharkey and Weise, 2015) leading to the generation of H_2_O_2_, which has been proposed to activate CEF (Strand et al., 2015). Results from a series of double mutants and inhibitors indicate that CEF in *hcef1* (Livingston et al., 2010a), and that seen in response to H_2_O_2_ (Strand et al., 2015), involves the chloroplast NDH complex and not the antimycin A sensitive FQR pathway.

In this work, we report on the isolation and characterization of *hcef2*, which was mapped to an unexpected locus involving tRNA editing. Despite very different processes involved, *hcef2* was found to have similar levels of CEF activation, H_2_O_2_ generation and increases in photoprotection as *hcef1*. This finding has strong implications for the role of CEF, including the possible involvement of reactive oxygen species in its regulation, and the critical importance of strict regulation of plastid proteome stoichiometries.

## Results

### Genetic selection of *hcef* mutants

As described in detail in Livingston et al. (2010a), we identified *hcef* mutants using a multi-stage selection process from a pool of ethyl methanesulfonate (EMS) mutagenized seeds (Columbia ecotype [Col-0], Lehle Seeds [M2E-02-05]). We first used chlorophyll fluorescence imaging to screen for plants that displayed high q_E_ phenotypes, likely indicating high light-induced *pmf*. We then subjected this population to secondary screening using a series of measurements of light-driven electron and proton transfer reactions, based on analysis of chlorophyll *a* fluorescence and absorbance changes (Sacksteder and Kramer, 2000; Baker et al., 2007; Baker, 2008; Livingston et al., 2010a) to identify mutants with elevated CEF.

### Growth of *hcef2*

**Figure 1:**
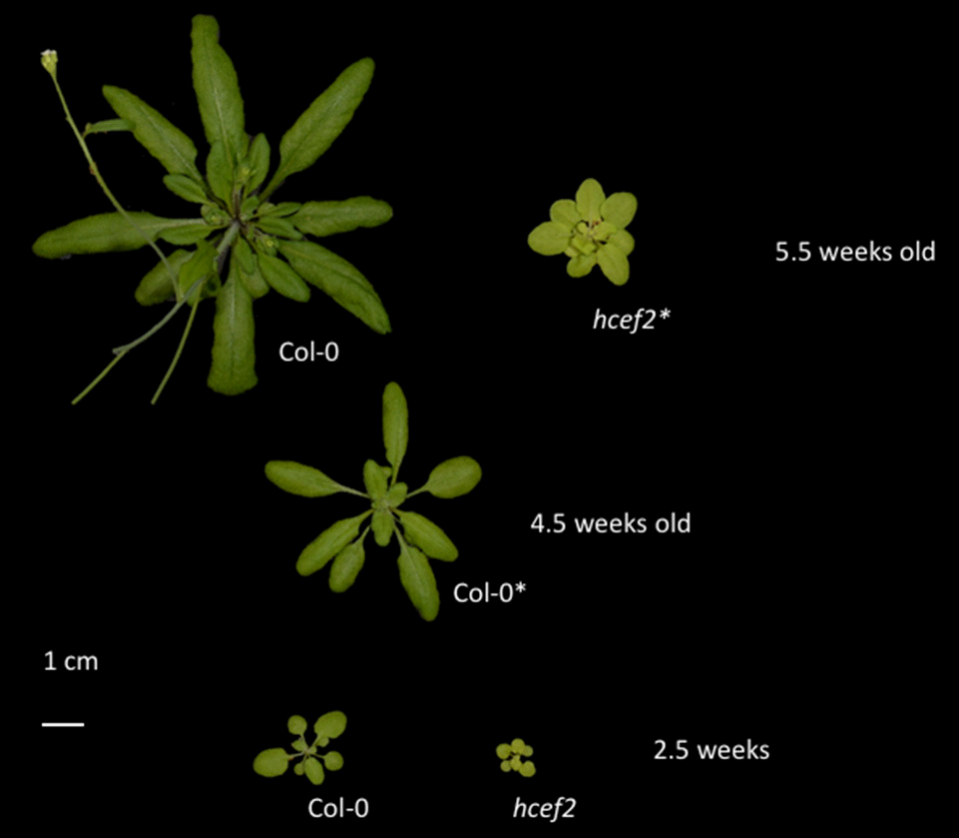
**Growth phenotype of Col-0 and ***hcef2*****. The developmental stage of each genotype used for experimentation is marked with (^*^).

The *hcef2* mutant grew photoautotrophically in soil, but with an impaired growth rate (Figure 1). Col-0 was fully expanded in 24–28 days, whereas *hcef2* of the same age had a rosette diameter <20% that of Col-0. Bolting was delayed in *hcef2*. The *hcef2* mutant displayed a slightly pale appearance owing to lower accumulation of chlorophyll compared to Col-0 levels per leaf area (157.8 mg/m^2^ ± 5.7 and 271.6 mg/m^2^ ± 5.1, respectively, *p* = 0.00001, Student's *t*-test, *n* = 3). All of the following results were obtained on plants at the same developmental stage, regardless of age, just prior to bolting.

### Photosynthetic properties of *hcef2* compared to Col-0 responses of photosynthetic electron transport

**Figure 2:**
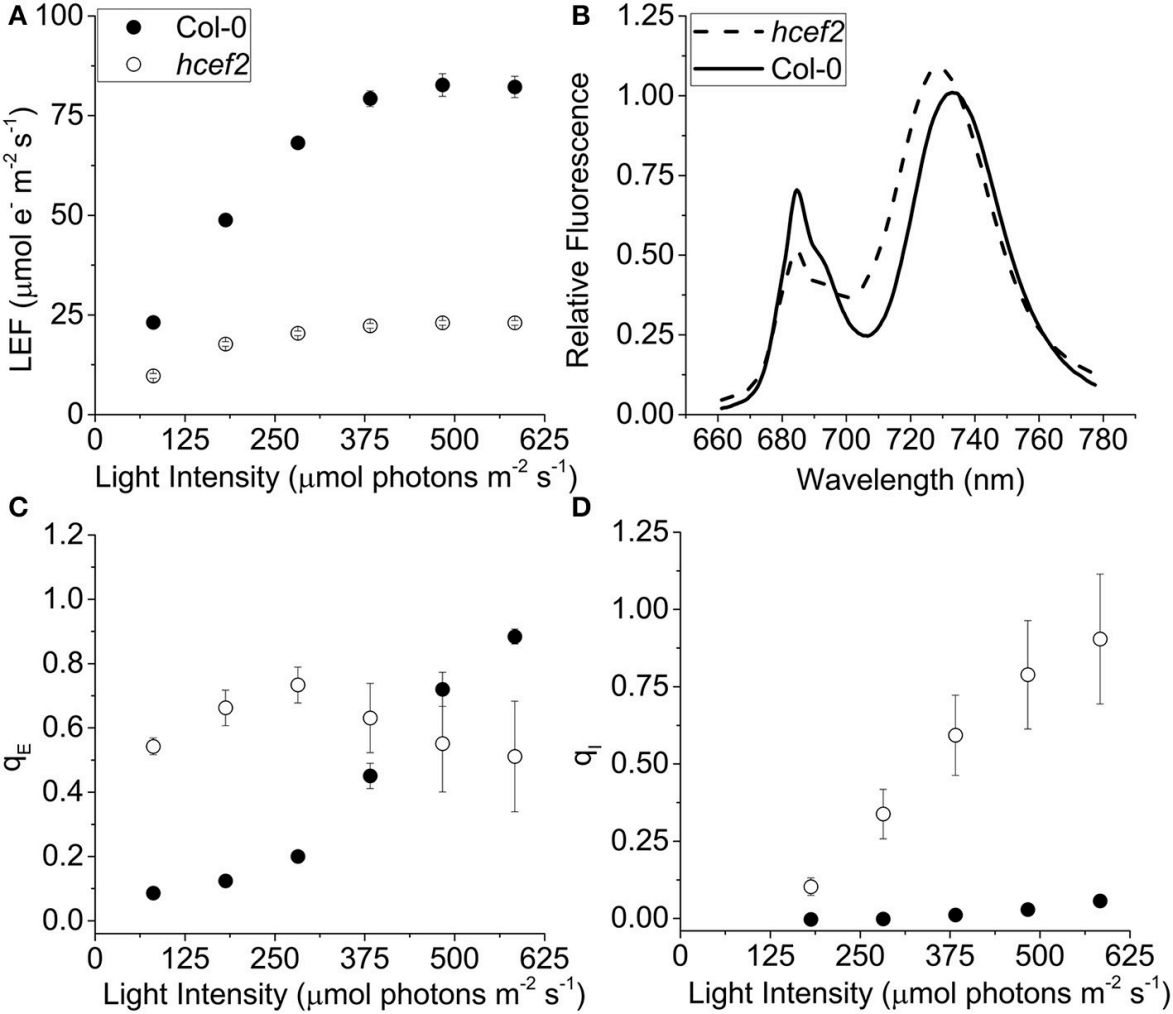
**Effects of ***hcef2*** on photosynthetic properties. (A)** Photosynthetic linear electron flow (LEF) as a function of light intensity. Col-0 (closed circles) and *hcef2* (open circles). Mean ± *SD, n* = 3. **(B)** 77k emission spectra. Col-0 (solid line) and *hcef2* (dashed line). Emission spectra are representative of three independent experiments. **(C)** Energy dependent exciton quenching (q_E_) as a function of light intensity. Col-0 (closed circles) and *hcef2* (open circles). Mean ± *SD, n* = 3. **(D)** Photoinhibition (q_I_) as a function of light intensity. Col-0 (closed circles) and *hcef2* (open circles). Mean ± *SD, n* = 3.

Chlorophyll *a* fluorescence was used to estimate linear electron flow (LEF) and q_E_ responses after accounting for differences in leaf absorptivity using the approach described previously (Dai et al., 1996; Livingston et al., 2010a). The *hcef2* mutant showed suppressed LEF rates across all light intensities used, about four-fold lower than Col-0 at saturating light (21.2 ± 1.68 and 82.6 ± 2.83 μmol e^−^ m^−2^ s^−1^, respectively, ~480 μmol photons m^−2^ s^−1^; Figure 2A). The half-saturation irradiance for LEF in *hcef2* (~90 μmol photons m^−2^ s^−1^) was ~65% that of Col-0 (~140 μmol photons m^−2^ s^−1^). In addition to differences in PSII electron transfer rates, Col-0 and *hcef2* showed distinct 77 K fluorescence emission spectra (Figure 2B), reflecting large changes in the composition, and state distribution of photosynthetic antenna complexes. The *hcef2* mutant showed a large increase in the relative emission of long-wavelength (~735 nm) emission associated with PSI antenna complexes, compared to shorter wavelength (685 nm) emission reflecting antenna complexes associated with PSII (for review see Krause and Weis, 1984). In addition, the PSI associated peak showed a strong blue shift, likely reflecting dissociation or loss of LCHI complexes from the PSI core (see below).

The maximal photochemical efficiency of PSII in dark-adapted leaves, estimated by the F_v_/F_M_ parameter, was substantially lower in *hcef2* (0.59 ± 0.066) compared to Col-0 (0.80 ± 0.003), probably indicating the accumulation of unrepaired photoinhibition in the mutant. Col-0 showed a typical sigmoidal response of q_E_ to light intensity (compared to results in e.g., Takizawa et al., 2007; Figure 2C), with an apparent half-saturation point at 375 μmol photons m^−2^ s^−1^ reaching ~0.9 at the highest light intensity tested, similar to previous results on plants grown under similar conditions (Livingston et al., 2010a). The q_E_ response of *hcef2* was distinct from those of Col-0, exhibiting high levels of q_E_ even at low light intensities. For example, at 80 μmol photons m^−2^ s^−1^, q_E_ in *hcef2* was approximately four-fold higher than in Col-0 (0.54 ± 0.026, 0.09 ± 0.12, respectively; Figure 2C). In *hcef2*, q_E_ reached a maximum at about 280 μmol photons m^−2^ s^−1^ with a value of 0.73 (±0.055, *n* = 3), but decreased slightly at higher irradiances likely reflecting the accumulation of photodamage at higher light in *hcef2*, as is consistent with the observed increase over this time in the slowly-reversible NPQ component, q_I_, which is typically associated with the onset of photoinhibition (Figure 2D).

### Responses of the photosynthetic proton circuit of *hcef2*

We measured dark interval relaxation kinetics (DIRK) of the electrochromic shift (ECS) to probe the proton circuit of photosynthesis (see reviews in Cruz et al., 2005; Baker et al., 2007). The extent of light-driven *pmf* was estimated from the total amplitude of the decay signal (ECS_t_); the relative rate of light-driven proton flux (*v*_H_^+^) was estimated from the initial slope of the ECS decay; and the conductivity of the thylakoid membrane to protons (*g*_H_^+^), which predominantly reflects the activity of the chloroplast ATP synthase, was estimated from the lifetime of the ECS decay (Sacksteder and Kramer, 2000; Cruz et al., 2005; Baker et al., 2007). From these values we calculated relative electron and proton fluxes through thylakoid components, and inferred the activation state of CEF (discussed in Livingston et al., 2010a,b; Strand and Kramer, 2014).

**Figure 3:**
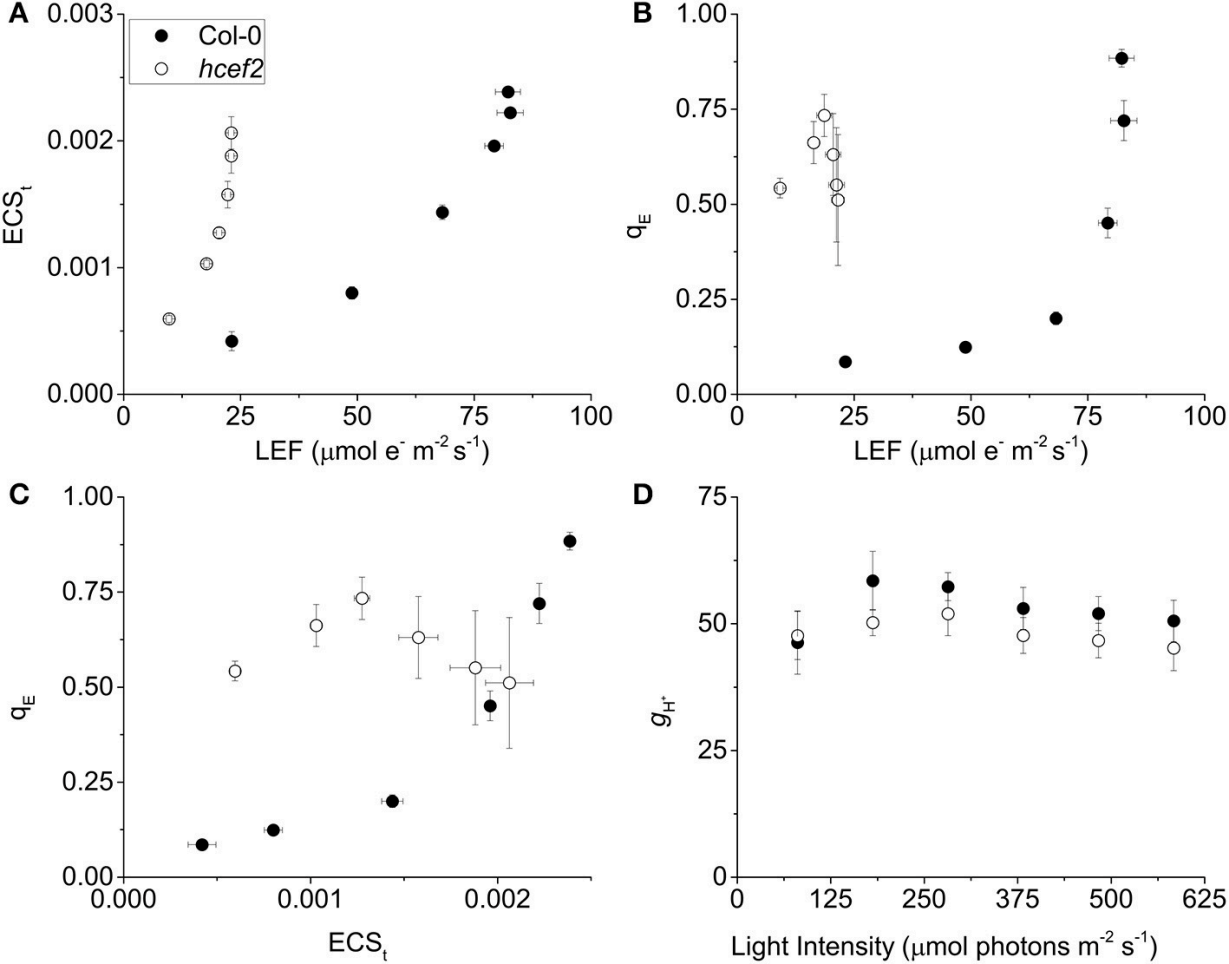
**Effects of ***hcef2*** on the photosynthetic proton circuit. (A)** Light driven transthylakoid *pmf* as measured by ECS_t_ as a function of LEF. Col-0 (closed circles) and *hcef2* (open circles). Mean ± *SD, n* = 3. **(B)** q_E_ as a function of LEF. Col-0 (closed circles) and *hcef2* (open circles). Mean ± *SD, n* = 3. **(C)** q_E_ as a function of ECS_t_. Col-0 (closed circles) and *hcef2* (open circles). Mean ± *SD, n* = 3. **(D)** Thylakoid proton conductivity (*g*_H_^+^) as a function of light intensity. Col-0 (closed circles) and *hcef2* (open circles). Mean ± *SD, n* = 3.

The *hcef2* mutant showed strongly decreased LEF compared to Col-0 (Figure 2A), yet produced substantially *higher* light-driven *pmf*, as indicated by increased ECS_t_-values as a function of LEF (Figure 3A). At an LEF-value of 20 μmol electrons m^−2^ s^−1^, *hcef2* had a four-fold higher ECS_t_ than Col-0 (0.002 ± 0.0001 and 0.0042 ± 0.000075, respectively, *n* = 3). The increased *pmf* was associated with qualitatively elevated q_E_ in *hcef2* (Figure 3B), as would be expected based on the lumen pH-dependence of the q_E_ response (reviewed in Müller et al., 2001). While Col-0 showed a sigmoidal dependence of q_E_ on ECS_t_, as previously reported (Takizawa et al., 2007), *hcef2* showed high activation of q_E_ at even low ECS_t_-values, but saturated at relatively low LEF or ECS_t_ extents (Figures 3B,C). The higher sensitivity of q_E_ responses in *hcef2* was more sensitive to estimated *pmf* changes (ECS_t_) in *hcef2* compared to Col-0, indicating additional factors beyond the *pmf* play a role in modulating the q_E_ response in *hcef2* (discussed below).

### Assessment of ATP synthase activity *in vivo*

As discussed earlier (Kanazawa and Kramer, 2002; Cruz et al., 2005), thylakoid *pmf* can be increased with respect to LEF by either accelerating proton influx through CEF or retarding proton efflux from the lumen by inactivating the chloroplast ATP synthase. To distinguish between these possibilities, we assessed the relative proton conductivity of the thylakoid membrane (*g*_H_^+^) using the ECS decay lifetime measurements. As shown in Figure 3D, *g*_H_^+^-values for Col-0 and *hcef2* were nearly identical, varying by <10%, indicating that the observed increases in *pmf* and q_E_ responses in *hcef2* could not be explained by down-regulation of the chloroplast ATP synthase.

### Estimates of CEF1 in *hcef2*

**Figure 4:**
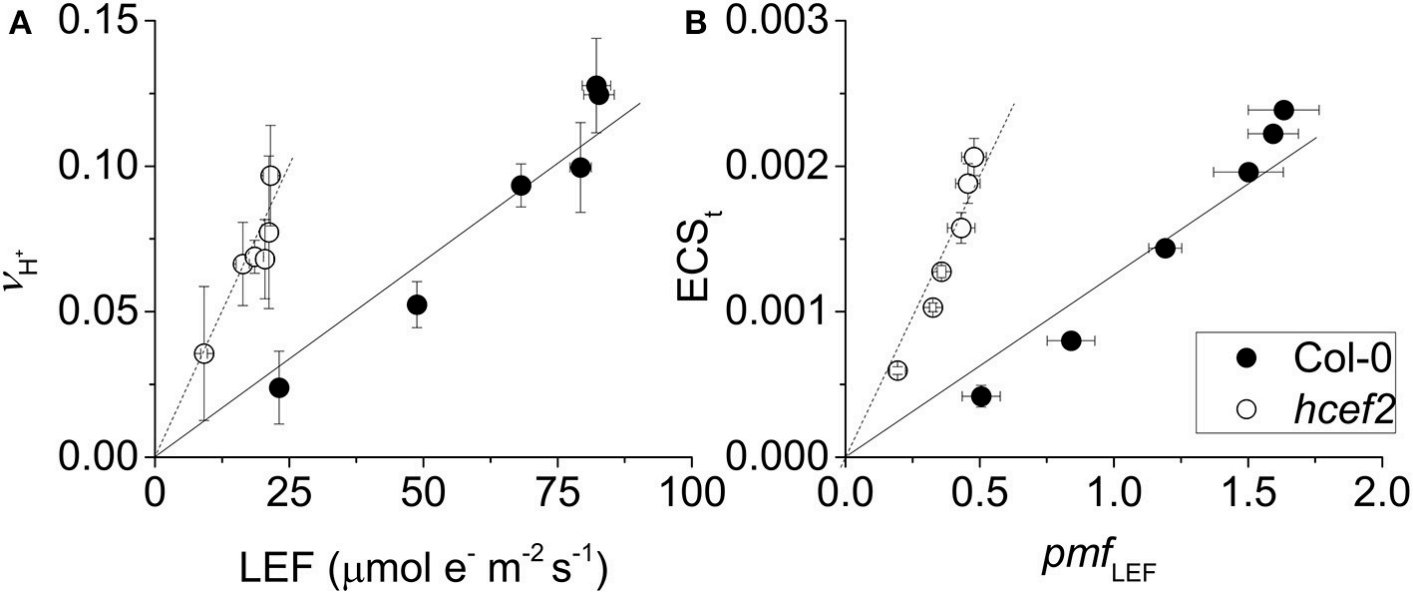
**Induction of CEF in Col-0 and ***hcef2***. (A)** Light driven transthylakoid proton flux (*v*_H_^+^) as a function of LEF. Col-0 (closed circles) and *hcef2* (open circles). Mean ± *SD, n* = 3. **(B)** ECS_t_ as a function of *pmf* generated by LEF (*pmf*_LEF_). Col-0 (closed circles) and *hcef2* (open circles). Mean ± *SD, n* = 3.

We next used three complementary approaches to assess the activation of CEF in *hcef2*. In the first approach, we compared proton flux estimated from initial decay rates of the ECS signal (*v*_H_^+^) with LEF estimated from chlorophyll *a* fluorescence parameters. When *v*_H_^+^ was plotted as a function of LEF (Figure 4A), *hcef2* showed a ~three-fold increase in slope over Col-0 (0.0039 ± 0.0011 and 0.0013 ± 0.0003 respectively, *p* = 0.0103 ANCOVA, *n* = 3), indicating an increase in the light-driven fluxes of protons over LEF. Because LEF produces a fixed H^+^/e^−^ stoichiometry, the additional protons would need to be supplied independently of PSII, i.e., by activation of CEF.

In the second approach (Figure 4B), we compared relative light-driven *pmf*, estimated by the ECS_t_ parameter, with calculations of the *pmf* from LEF alone (*pmf*_LEF_; Avenson et al., 2005). This approach is based on different assumptions than the first, and is largely independent of extrinsic factors, such as the leaf content of ECS-responding carotenoids, etc. (see discussion in Avenson et al., 2004; Baker et al., 2007). The dependence of ECS_t_ against *pmf*_LEF_ was approximately three-fold higher in *hcef2* compared to Col-0 (0.0037 ± 0.0005 and 0.0012 ± 0.0003, respectively, *p* < 0.0001 ANCOVA *n* = 3), indicating that *hcef2* accumulates larger extents of *pmf* than can be attributed to changes in LEF, supporting the conclusion that CEF is strongly activated in *hcef2*. It should be noted that estimates of LEF by analysis of chlorophyll fluorescence depend on the fraction of light energy absorbed by PSII. The 77K fluorescence emission spectra (Figure 2B) show a decrease in the relative fluorescence of PSII at 685 nm relative to that attributable to PSI at about 735 nm, possibly indicating a decrease in PSII relative to PSI excitation, but the potential error introduced by this antenna change should lead to an underestimation of the increase in CEF/LEF for the data in Figures 4A,B.

**Figure 5:**
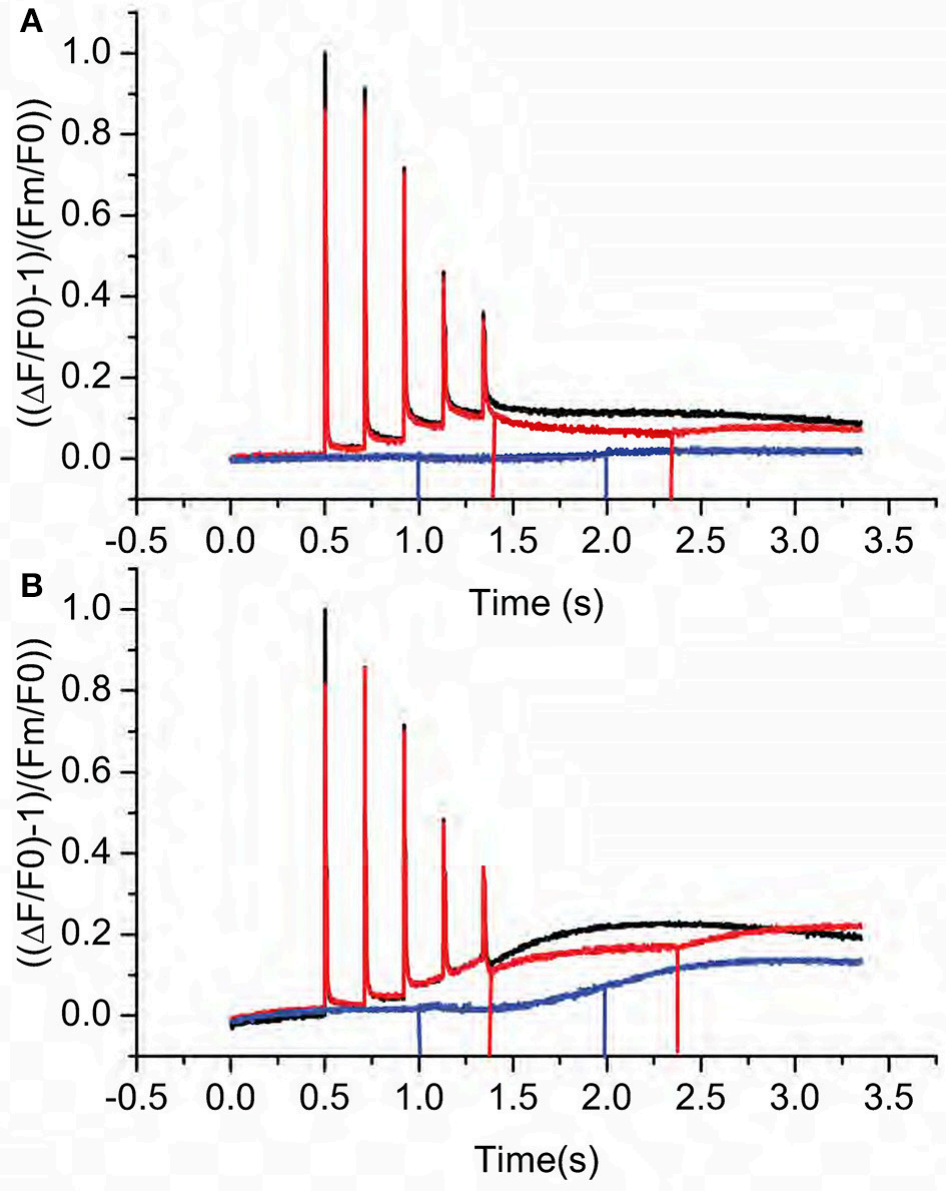
**Kinetics of post-illumination fluorescence rises in Col-0 and ***hcef2***. (A)** Relative fluorescence yield changes in Col-0 in the dark after a series of actinic flashes (black), changes with far-red illumination after the actinic flashes (red), and changes with far-red illumination in the dark. Data is normalized using F0 and Fm-values from the first data set (black) and is representative of three independent experiments. Lines indicate the start and stop of far red illumination, respectively. **(B)** Relative fluorescence yield changes in Col-0 in the dark after a series of actinic flashes (black), changes with far-red illumination after the actinic flashes (red), and changes with far-red illumination in the dark. Data is normalized using F0 and Fm-values from the first data set (black) and is representative of three independent experiments. Lines indicate the start and stop of far red illumination, respectively.

In the third approach, we measured post-illumination changes in chlorophyll *a* fluorescence that indicate the non-photochemcial reduction of the PQ pool associated with activation of the NDH-pathway for CEF (Burrows et al., 1998; Sazanov et al., 1998; Shikanai et al., 1998; Gotoh et al., 2010). Typically, such fluorescence rise experiments are conducted by exposing leaves to continuous illumination for a few minutes and the fluorescence yield is followed after switching off the light. An initial decrease of fluorescence is caused by rapid oxidation (on tens to hundreds of milliseconds time scale) of Q_A_ by PQ. When NDH is active, this initial decay phase is followed by a slower fluorescence rise as PQ becomes progressively reduced by NDH. During initial trials, we found that the decay and rise phases was more clearly resolved when leaves from growth conditions were partially dark adapted (for 10 min) and exposed to short (10 ms duration) pulses of intense actinic light. As shown in Figure 5A, each pulse resulted in increased fluorescence yield reflecting light-induced reduction of Q_A_. The fluorescence yield then deceased in multiple phases after each flash. A rapid phase, with a half time of less than a few ms, reflected the equilibration of Q_A_ and PQ redox states in the dark. In Col-0 (Figure 5A, black line), each pulse resulted in progressively more reduced PQ pool as indicated by the increases in dark fluorescence levels. This interpretation was confirmed by the decreased in fluorescence yield induced by far red (730 nm) illumination (Figure 5A, red line), which preferentially excites PSI photochemistry resulting in net oxidation of the PQ pool and Q_A_. Cessation of far-red illumination resulted in a slow return to higher fluorescence yields indicating reduction of the PQ by a non-photochemical process, most likely through a process related to CEF. These phenomena were also observed in *hcef2* (Figure 5B, black line), but were stronger and more rapid. In fact, a distinct fluorescence rise phase was seen after the third flash in *hcef2* that we interpret as indicating strong activation of PQ reductase activity. The interpretation was confirmed by application of far-red illumination (Figure 5B, red line), which resulted in substantial quenching of the signal. Rise occurred after each additional flash and continued during the following dark period. We conclude that *hcef2* has a substantially higher activity of PQ reductase than Col-0. In addition, in the dark, application of far-red illumination only minimally quenched basal fluorescence in both Col-0 and *hcef2* (Figure 5, blue lines) indicating F0-values were not strongly affected by pre-reduction of QA. However, in *hcef2*, there was a rise in fluorescence after application of far-red (Figure 5B, blue line), suggesting stimulation of PQ reduction that was not seen in Col-0 (Figure 5A, blue line).

### Antimycin A infiltration of *hcef2*

**Figure 6:**
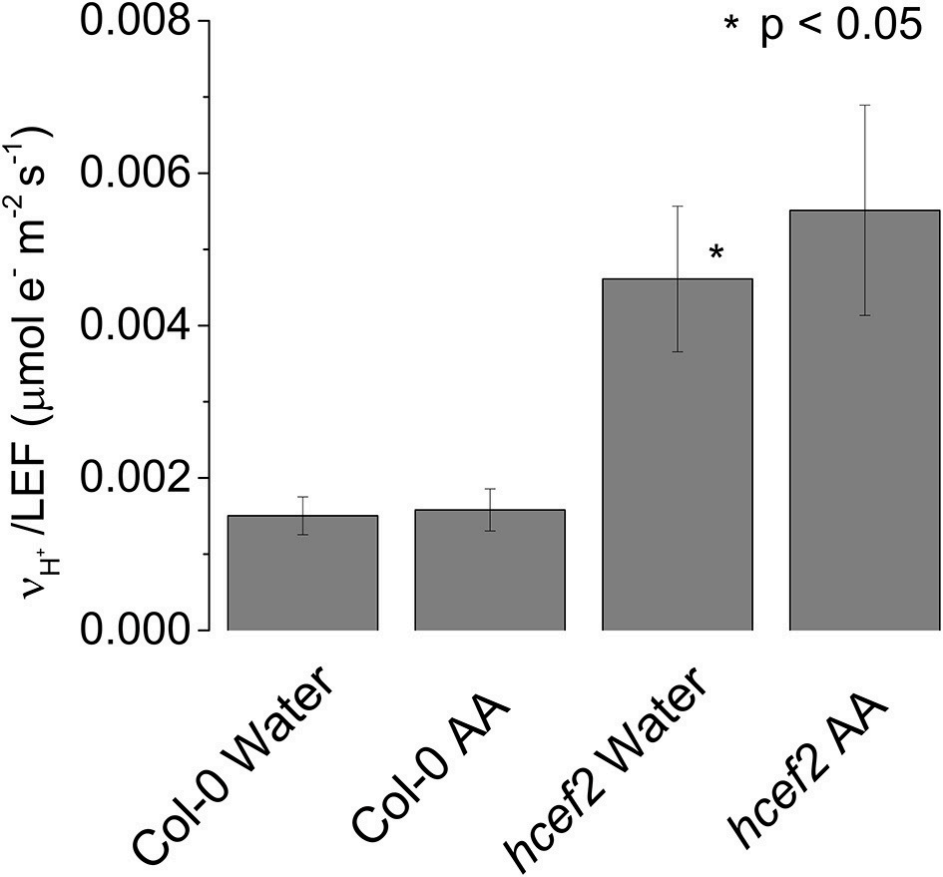
**Antimycin a insensitivity of CEF in ***hcef2*****. Slope of *v*_H_^+^/LEF in leaves infiltrated with either water or antimycin A (AA). Mean ± *SD, n* = 3.

In Col-0 we observed no significant differences in the ratio of *v*_H_^+^/LEF between leaves infiltrated with water or 20 μM antimycin A (0.0015 ± 0.0025 and 0.00158 ± 0.00026 respectively *p* > 0.05 *n* = 3, Figure 6). The elevated ratio of *v*_H_^+^/LEF in *hcef2* was also unaffected by 20 μM antimycin A (0.00551 ± 0.0014 and 0.00461 ± 0.00095, respectively *p* > 0.05 *n* = 3, Figure 6), indicating *hcef2* CEF is antimycin A insensitive and thus does not occur through the antimycin A-sensitive CEF pathway.

### Mapping the genetic locus of *hcef2* as TADA1

**Figure 7:**
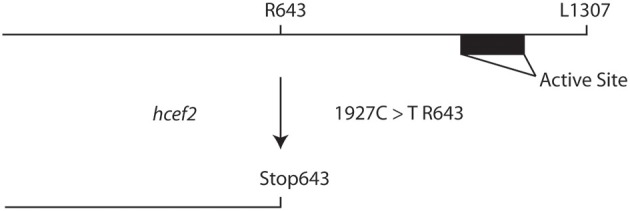
**Introduction of a stop codon into the TADA1 locus in ***hcef2*****. Translation is terminated before the active site of TADA1, leading to a loss of function.

**Figure 8:**
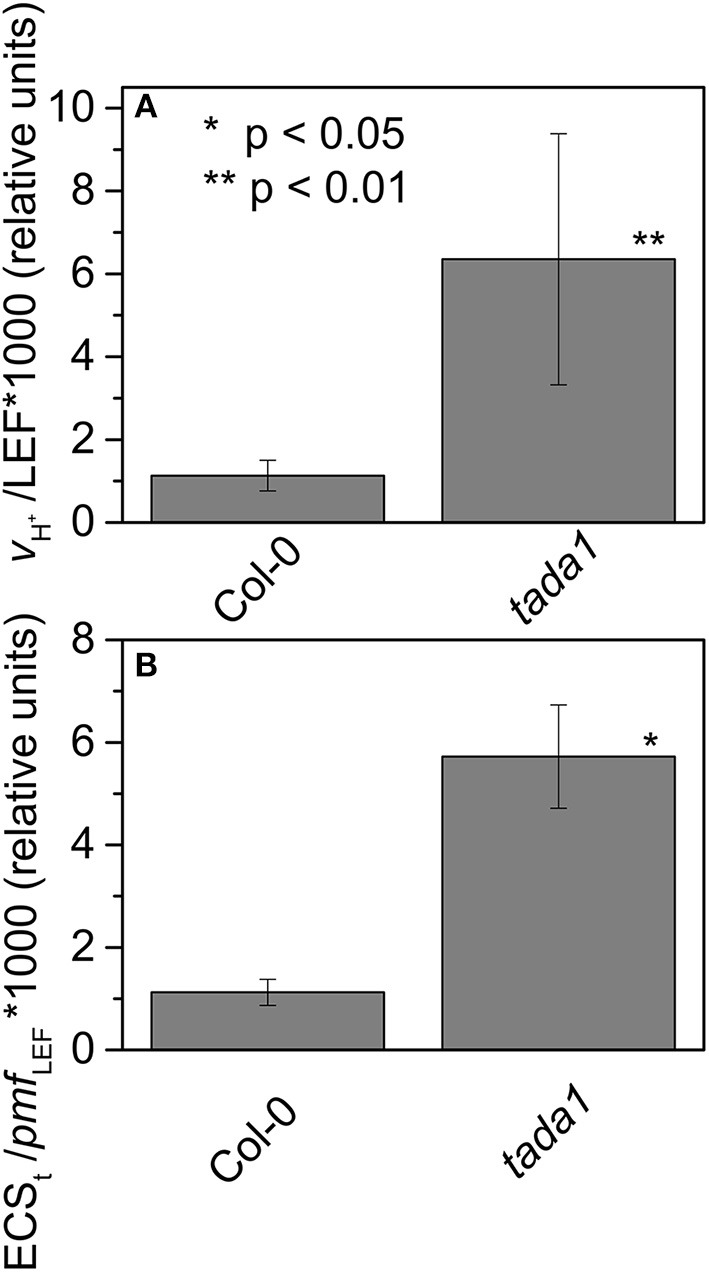
**Elevated CEF in ***tada1*****. The ratios of *v*_H_^+^/LEF **(A)** and ECS_t_/*pmf*_LEF_ **(B)** of intact leaves were measured and estimated as described in Section Methods and Figure 4. Date represent the mean ± *SD* with *n* = 3.

Map based cloning and deep sequencing was used to identify the probable genetic locus for the *hcef2* mutation to a point mutation in TADA1 (At1G68720). This *C* > *T* mutation introduces a stop codon at R643 (Figure 7), eliminating the C-terminus of the protein containing the active site required for function (Delannoy et al., 2009; Karcher and Bock, 2009). The T-DNA insert line GK-119G08 contains an insertion in the first exon in At1G68720 (Delannoy et al., 2009). Similar to *hcef2, tada1* showed strongly increased CEF as indicated by a five-fold increase relative to Col-0 of *v*_H_^+^/LEF (0.0063 ± 0.003 and 0.0011 ± 0.0004, respectively, *p* < 0.05 *n* = 3; Figure 8A) as well as in ECS_t_/*pmf*_LEF_ (0.0057 ± 0.001 and 0.0011 ± 0.0003, respectively, *p* < 0.001, *n* = 3; Figure 8B) relationships. These results indicate increased CEF equal to or greater than *hcef2* and support the identification of *hcef2* mutation within TADA1. Likewise, *tada1* showed no statistical difference from *hcef2* in leaf chlorophyll content or absorptivity.

**Figure 9:**
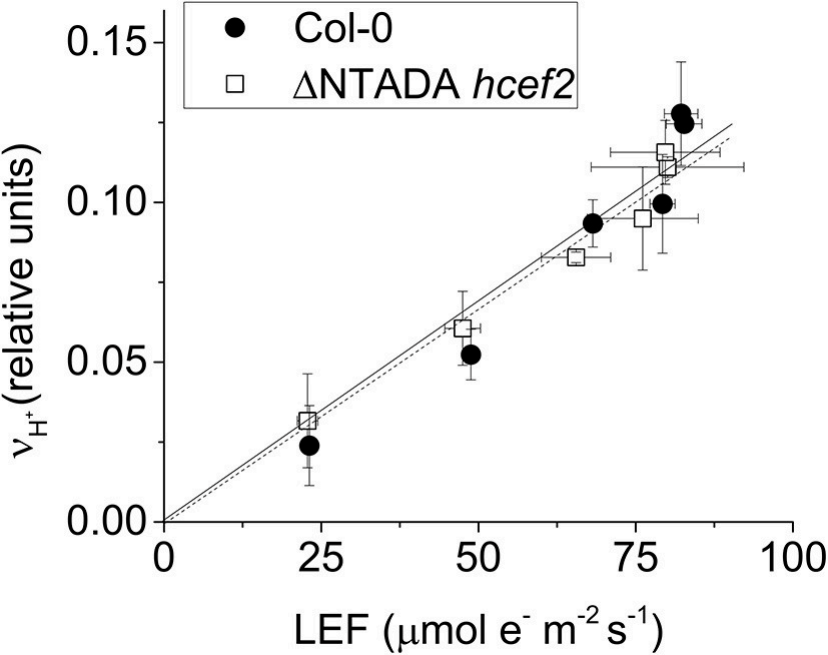
**TADA1 complementation of ***hcef2*****. *v*_H_^+^/LEF of intact leaves of Col-0 (filled circles) and *hcef2* P35S:ΔN TADA1 (open squares). Mean ± *SD, n* = 3.

Delannoy et al. (2009) showed that the *tada1* phenotype was reversed by expressing the C-terminus of the tada1 (At1G68720) behind a 35S promoter. We transferred this construct by crossing the complimented *tada1* mutant with *hcef2* followed by segregation and genotyping for homozygocity of the *hcef2* mutation, lack of the *tada1* insertion, and possession of the P35S:ΔNTADA1 construct. Verified lines were analyzed spectroscopically for suppression of the *hcef2* phenotype. The increased *v*_H_^+^ as a function of LEF seen in *hcef2* (Figure 4A) was completely suppressed in the *hcef2* P35S:ΔNTADA1 line (Figure 9), i.e., the slope returns to Col-0-values (0.0013 ±.00029 and 0.0013 ± 0.00031, respectively, *p* > 0.05, ANCOVA *n* = 3). These results confirm TADA1 as the site of the mutation causing elevated CEF in *hcef2*.

### Translational defects lead to increases in CEF

**Figure 10:**
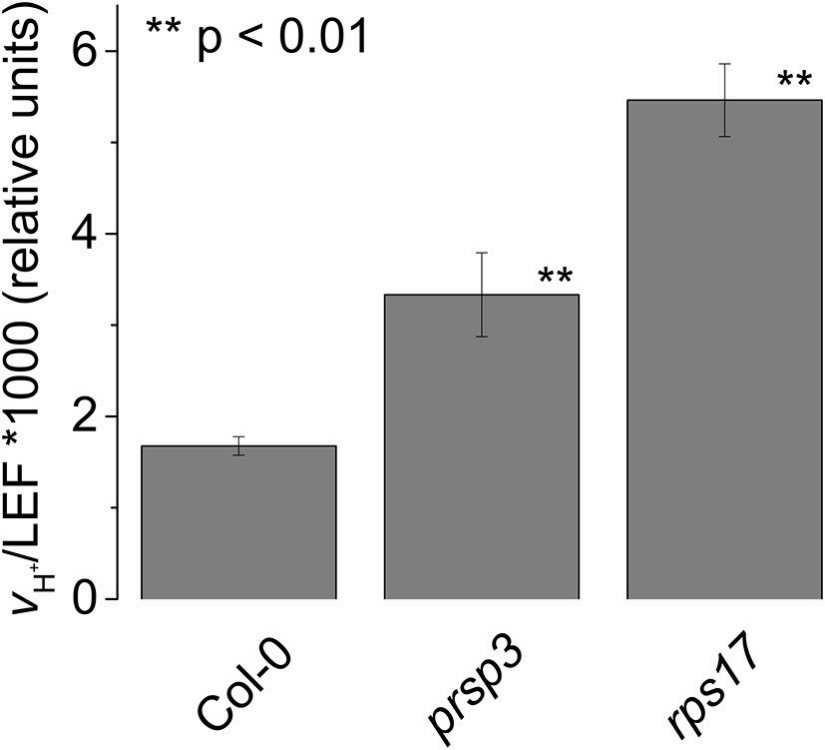
**Elevated CEF in the ribosomal mutants ***prsp3*** and ***rps17*****. *v*_H_^+^/LEF of intact leaves. Mean ± *SD, n* = 3.

The TADA1 gene codes for a tRNA editing enzyme, suggesting that a defect in translation machinery somehow leads to increased CEF. To test if this effect is a general consequence of decreased chloroplast translation efficiency, we assayed for increased CEF in mutants defective in nuclear encoded peripheral ribosomal proteins. The *prsp3-1* mutant contains a T-DNA insert in the At1g68590 locus with a complete loss of PRSP3 (Tiller et al., 2012). The *rps17* mutant contains a T-DNA insert in the At1g79850 locus, resulting in decreased expression of RPS17 by 85% (Tiller et al., 2012). Both of these mutations resulted in partial loss of ribosomal proteins and impaired chloroplast translation (Tiller et al., 2012). The extents of CEF as measured by *v*_H_^+^/LEF were increased by about two-fold compared to Col-0 in both *prsp3-1* (0.0033 ± 0.0005 and 0.0017 ± 0.0001, respectively, *p* < 0.001, *n* = 3; Figure 10), and *rps17* (0.0017 ± 0.0001 and 0.0055 ± 0.0004, respectively, *p* < 0.001, *n* = 3). These results suggest that elevated CEF may be a general response to disruption of chloroplast translation.

### Flash induced relaxation kinetics in dark-adapted leaves of *hcef2*

**Figure 11:**
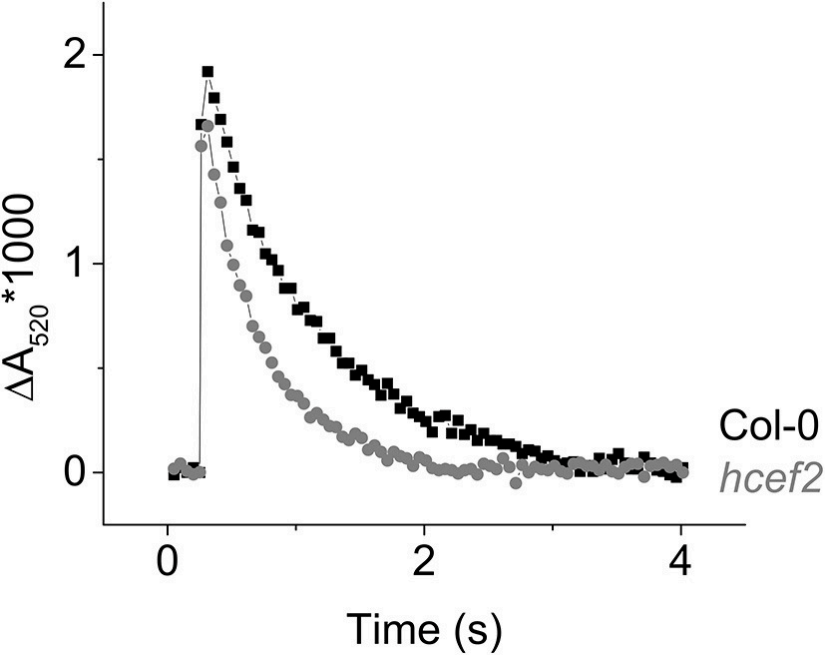
**Flash induced relaxation kinetics of the electrochromic shift in Col-0 (black) and ***hcef2*** (gray) leaves infiltrated with 100 μM methyl viologen**. Data is representative of three independent experiments.

The decay of the flash induced ECS signal has been used to monitor the generation of electric field (Δψ) across the thylakoid and its dissipation by the movements of protons through the ATP synthase or counterions through ion channels (Kramer and Crofts, 1989). In dark-adapted leaves, or leaves infiltrated with methyl viologen (Figure 11), the ATP synthase becomes inactivated by oxidation of regulatory thiols, slowing the decay of the ECS signal. The residual decay, measured at low flash intensity to prevent re-activation of ATP synthase, reflects leakage of protons and counterions across the thylakoid membrane. In Col-0, this residual decay was slow, with a lifetime of about 0.8 s, similar to previous results (Kramer and Crofts, 1989). A substantially increased ECS decay rate was about two-fold faster in *hcef2* (lifetime = ~0.4 s, Figure 11), indicating an increased rate of proton or ion leakage from the lumen.

### Spectral analyses of thylakoid cytochrome composition in Col-0 and *hcef2*

**Figure 12:**
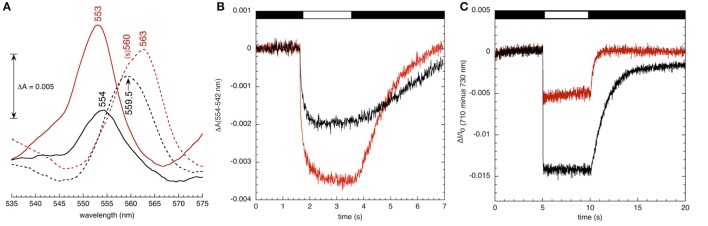
**(A)** Redox difference visible absorption spectra of the cytochrome bf complex in broken chloroplast preparations from Col-0 (black) and *hcef2* (red). Broken chloroplasts from Col-0 and *hcef2* were prepared as described and resuspended to a chlorophyll concentration of 150 μg/ml in an assay buffer consisting of 10 mM HEPES (pH 7.6), 5 mM MgCl_2_ and 0.2% (w/v) dodecyl maltoside. Difference spectra for cytochrome *f* (solid line, 0.5 mM ascorbate reduced *minus* 0.5 mM potassium ferricyanide oxidized) and cytochrome *b* (broken line, dithionite reduced *minus* 8 mM ascorbate reduced) are shown. Difference spectra were obtained at room temperature and averaged from four scans per redox condition per sample with a spectral resolution of 1 nm. A five point smoothing function was applied to the data, and spectra were adjusted for dilution effects. Peak positions are labeled. “(s)” refers to shoulder. Spectra of PSII-associated cytochrome b-559 (obtained from [(8mM ascorbate – 0.5 mM ferricyanide) *minus* (0.5 mM ascorbate – 0.5 mM ferricyanide)] are not shown. **(B)** Oxidation-reduction kinetics of cytochrome *f* in Col-0 (black) and *hcef2* (red) thylakoids. Kinetics were measured in broken chloroplast preparations at a chlorophyll concentration of 50 μg/mL^−1^. The assay buffer consisted of 10 mM HEPES (pH 7.6), 5 mM MgCl_2_, 20 mM KCl, 10 μM DCMU, 1 mM methyl viologen, 10 μM valinomycin, 10 μM nigericin, and 2 mM ascorbate. Red actinic light (620 nm) was applied at an intensity of 200 μE m^−2^ s^−1^ (indicated by white bar in figure). Measuring pulses were provided by narrow-band filtered LEDs at 554- and 545-nm. The (dark) interval between measuring traces was 30 s, and eight traces were averaged per experiment. **(C)** P_700_ content in isolated thylakoids **(A)** 710 nm *minus* 730 nm absorbance changes in Col-0 (black) and hcef2 (red) thylakoid preparations The chlorophyll concentration was 50 μg Chl · ml^−1^, and the assay buffer consisted of 10 mM HEPES (pH 7.6), 5 mM MgCl_2_, 2 mM ascorbate, 1 mM methyl viologen, and 10 μM DCMU. Hydroxylamine (added immediately prior to data collection) was present at 1 mM to remove any PSII-associated variable fluorescence. Red actinic light (620 nm) was applied at an intensity of 200 μmoles photons m^−2^ s^−1^ (indicated by white bar in figure). The (dark) interval between measuring traces was 60 s, and four traces were averaged per experiment.

To assess effects of *hcef2* on thylakoid cytochrome composition, we performed a rough redox titration analysis while monitoring the absorbance spectrum between 535 and 575 nm, using difference spectra (Figure 12A) of the 0.5 mM ascorbate reduced *minus* 0.5 mM potassium ferricyanide oxidized samples and the difference spectra of dithionite reduced *minus* 8 mM ascorbate reduced samples were used as indicators of the relative contents of cytochromes *f* and *b*. In wild type chloroplasts, the reduced-minus oxidized difference spectrum around 554 nm reflects cytochrome *f*, whereas lower potential absorbance spectra in the 560–565 nm region contains overlapping contributions from at least three heme *b* species, including the PSII cytochrome *b*_559_ (at around 560 nm) and cyt *b*_H_ and *b*_L_ (near 563 nm) of the *bf* complex. In Col-0 thylakoids the dithionite-ascorbate absorbance difference spectrum in the heme *b* region was approximately double that in the ascorbate-ferricyanide cytochrome *f* region consistent with what is expected for the native complex (i.e., containing two cytochrome *b* hemes for each cytochrome *f*). It should be noted that the cytochrome *b* spectrum was broader (full width at half height of 12 nm) than expected if only cytochrome *b*_H_ and *b*_L_ contributed, likely indicating at least some contributions to the spectrum from cytochrome *b*_559_, though this should not have dramatically affected the estimated ratio of cytochrome *b* to cytochrome *f* (see discussion in Kramer and Crofts, 1994). The ratios of cytochrome *b* and *f* spectra were strongly affected by the *hcef2* mutation, with a larger cytochrome *f* signal relative to cytochrome *b* signal, suggesting that the ratio of heme *f* to heme *b* was increased in the mutant by a factor of at least two, and was accompanied by an apparent shift of 1 nm to the blue in the α-band of the cytochrome *f* redox difference spectrum of *hcef2* compared to Col-0, possibly indicating a modification of the protein environment around heme *f* in the mutant. In addition, the shape of the cytochrome *b* region became more asymmetric, and shifted to the red suggesting changes in the ratios of contributions from cytochrome *b*_559_ and cytochrome *b*_H_.

As shown in Figure 12B, illumination of uncoupled thylakoids in the presence of 10 μM DCMU, 2 mM ascorbate, and 1 mM methyl viologen resulted in absorbance changes at 554–545 nm, attributable to oxidation of cytochrome *f*. Interestingly, the extent of cytochrome *f* oxidation signal was almost two-fold larger in *hcef2* than Col-0, suggesting that the increased content of cytochrome *f* was photooxidizable through redox interactions with PC.

### PSI content and P_700_ redox status

Figure 12C shows light-induced P_700_ redox changes measured by absorbance changes at 710–730 nm in thylakoid preparations of *hcef2* and Col-0 treated with DCMU and hydroxylamine to inhibit PSII electron transfer and variable fluorescence, as well as methyl viologen as a final electron acceptor, allowing us to photoaccumulate essentially all P_700_ in its oxidized state. The extents of the photo-induced bleaching (which reflect the disappearance of the reduced P_700_ state) indicate that *hcef2* had <50% the level of active PSI complexes.

**Figure 13:**
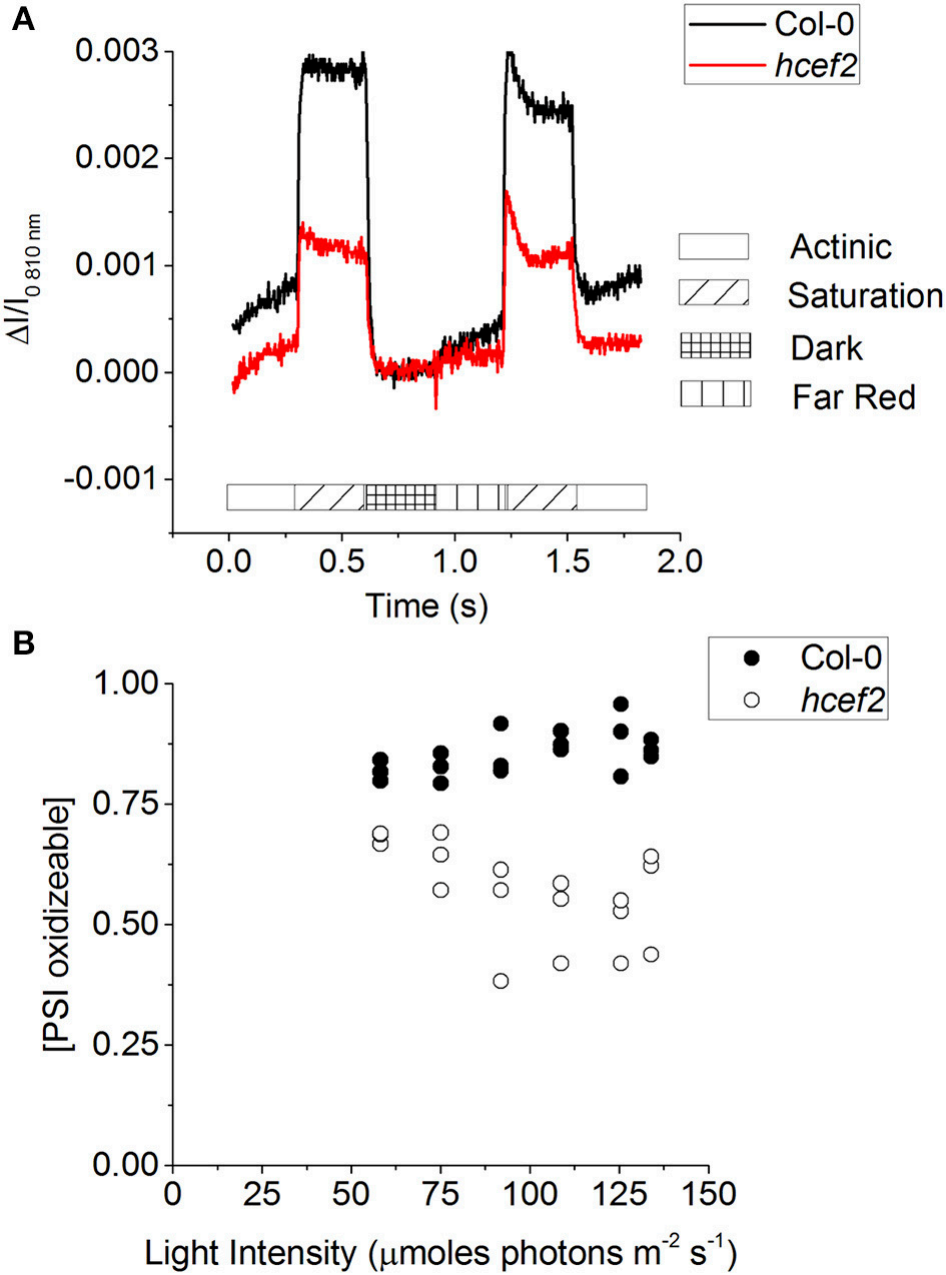
**P_**700**_ redox status. (A)** From the steady state (135 μmoles photons m^−2^ s^−1^) A saturation flash was applied to oxidize PSI, followed by a dark interval to allow PSI to be reduced. Far red illumination was applied to preferentially excite PSI and open up the acceptor side, followed by another saturation flash to fully oxidize PSI. **(B)** The fraction of oxidizable PSI in Col-0 (closed circles) and *hcef2* (open circles) calculated from **(A)** as described in Klughammer and Schreiber (1994).

Figure 13A shows saturating pulse induced absorbance changes at 810 nm in intact leaves, which indicates the appearance of the P_700_^+^ state, during a series of light-pulses to indicate the redox status of PSI in the steady-state (after 10 min of illumination; based on the procedure presented in Klughammer and Schreiber, 1994). First, data was taken at the start of the curves to probe the redox state of P_700_ during steady-state actinic illumination. A strong saturating pulse was then applied to determine the number of photo-oxidizable PSI centers, i.e., those with reduced P_700_ and oxidized F_A_/F_B_ electron acceptors. Compared to Col-0, *hcef2* showed about 50% fewer such centers. After the saturating pulse, the actinic light was switched off to allow all P_700_^+^ to go reduced. The difference in the signal from the baseline to the dark indicates the total number of PSI centers oxidized during steady-state illumination, which was substantially lower in hcef2 than Col-0. Following the dark interval, the leaves were exposed to far-red illumination to oxidize electron carriers between PSI and PSII, followed by a second, saturation pulse to essentially fully oxidize P_700_. The extents of oxidation during the second pulse were consistent with the results in Figure 13A, showing an ~50% decrease in active PSI centers in *hcef2* compared to Col-0. The ratio of peak signals during first and second saturation pulses is used as an indicator of the degree of reduction of the PSI electron acceptor pool during steady state illumination. Along with being significantly more reduced, the oxidizable fraction is also decreased in *hcef2* (Figure 13B), this is likely due to the closure of the acceptor side of PSI.

### Thylakoid protein levels in *hcef2*

**Figure 14:**
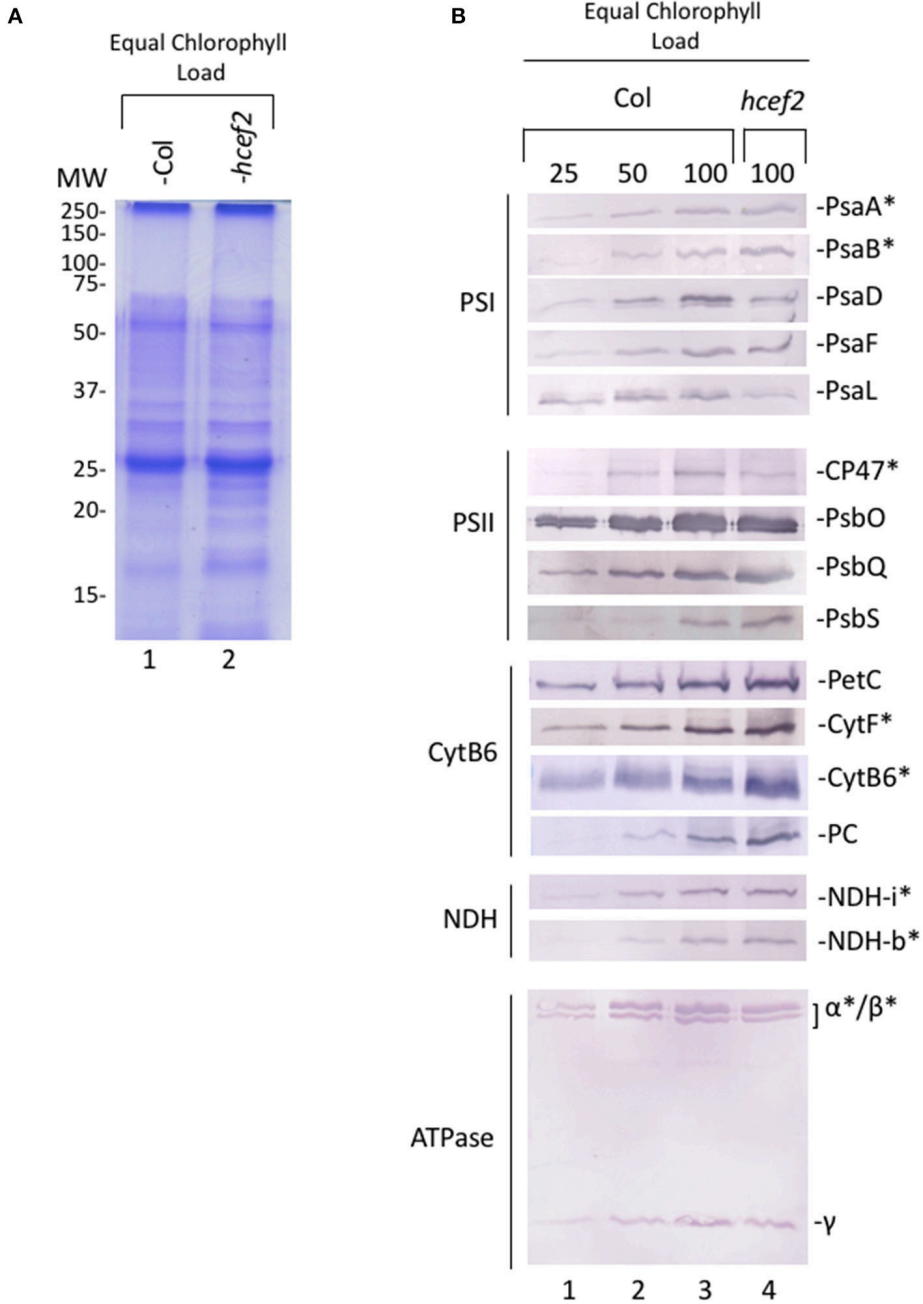
**Photosynthetic protein accumulation in Col-0 and ***hcef2*** thylakoid membranes. (A)** Coomassie stain of thylakoid proteins separated by SDS-PAGE and **(B)** western blot of thylakoid electron and proton transport chain components. Chloroplast encoded proteins are denoted by (^*^).

Figure 14 shows protein levels of thylakoids isolated from Col-0 and *hcef2* and separated by SDS-PAGE on a chlorophyll basis. Multiple antibodies were used against subunits of the thylakoid complexes to look for changes in subunit stoichiometries.

PSII subunits PSBO and PSBQ accumulated in *hcef2* at Col-0 levels, while CP47 was about half that of Col-0. PSBS was seemingly increased in *hcef2* over Col-0, possibly explaining the increased q_E_ sensitivity to ECS_t_ in *hcef2* (Li et al., 2002), but not ruling out the possibility of altered ΔpH/*pmf* (discussed in Cruz et al., 2005). The *bf* complex was overall increased in *hcef2*, in agreement with increase in chromophores seen in the redox cut (Figure 12A). NDH subunits levels did not increase, contrary to previously described *hcef* mutants (Livingston et al., 2010a; Strand et al., 2015), however this is not without precedence (Gotoh et al., 2010). The levels of ATP-γ and ATP-α/β subunits of the ATP synthase also accumulated to Col-0 levels in *hcef2*, suggesting that the ion leak in seen in *hcef2* (Figure 11) is a phenomenon not related to stoichiometry changes of subunits within the ATP synthase.

The core subunits of PSI (PSAA and PSAB), as well as PSAF, were similar to wildtype levels. However, PSAD accumulated to only 50% of Col-0 levels, while PSAL accumulation appeared to be even lower. Thus, the loss of functional PSI centers, seen in Figures 13A,B), probably reflects the loss of the smaller subunits of the complex, implying that a substantial fraction of PSI proteins are not assembled into active complexes.

### The *hcef2* and related mutants show elevated rates of H_2_O_2_ production

**Figure 15:**
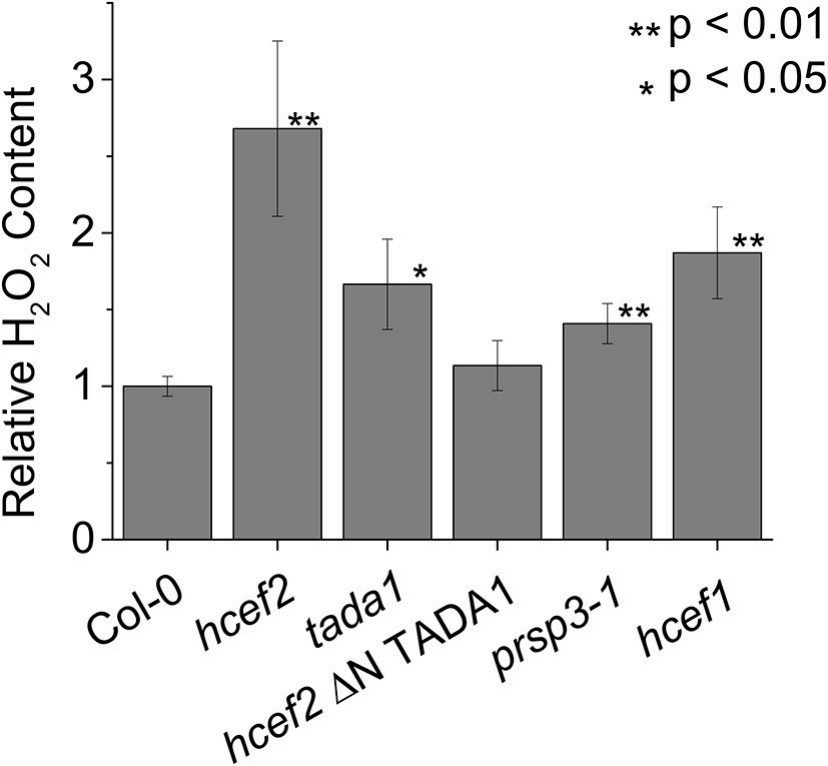
**Relative accumulation of H_**2**_O_**2**_ in Col-0 and mutant lines**. Leaf H_2_O_2_ content was determined using resorufin fluorescence resulting from the reaction of Amplex red with H_2_O_2_. Fluorescence was expressed on a chlorophyll basis and normalized to the mean Col-0 fluorescence level. Bars represent mean ± *SD, n* = 3.

Figure 15 shows relative leaf H_2_O_2_ content in Col-0, *hcef2, tada1, hcef2* P35S:ΔNTADA1, *prsp3*, and *hcef1*. Both *hcef2* and *tada1* had significantly higher H_2_O_2_ accumulation than Col-0 (2.67 ± 0.57, 1.67 ± 0.29, and 1.00 ± 0.06, respectively, *p* < 0.01 and *p* < 0.02, respectively, *n* = 3) while the complimented line, *hcef2* P35S:ΔNTADA1, had H_2_O_2_ levels similar to Col-0 (1.13 ± 0.16 *p* = 0.25, *n* = 3). The plastid ribosomal mutant *prsp3* also had a significantly higher level of H_2_O_2_ (1.41 ± 0.13, *p* < 0.01, *n* = 3). In addition, the *hcef1* mutant also was shown to accumulate increased levels of H_2_O_2_ than Col-0 (1.87 ± 0.30 *p* < 0.01, *n* = 3).

## Discussion

### Disruption of protein translation in *hcef2* leads to activation of CEF involving the chloroplast NDH complex

The *hcef2* mutant was identified by progressively screening mutants using a series of criteria designed to identify mutants with elevated CEF. Several independent lines of evidence support strong activation of CEF in *hcef2*, including comparison of proton and electron fluxes (Figure 4A), *pmf* related parameters (Figure 4B), post-illumination fluorescence yield changes (Figure 5), and reduction of P_700_^+^ in the presence of DCMU. The elevated CEF in *hcef2* was found to be insensitive to antimycin A (Figure 6), arguing against the participation of the FQR pathway (Cleland and Bendall, 1992; Munekage et al., 2004), and in favor of the NDH complex, as previously shown for *hcef1* (Livingston et al., 2010a).

Surprisingly, we mapped *hcef2* to a non-sense mutation in the Tada1 locus, leading to a deletion of the C-terminal active site of TADA1, an enzyme that modifies the chloroplast arginine tRNA^ACG^ to tRNA^ICG^. Loss of TADA1 leads to the use of the less efficient “two out of three” codon recognition mechanism (Delannoy et al., 2009).

While the effect of the *hcef2* (*tada1*) mutation should affect the translation of ~80% of the chloroplast-encoded genes (Sato et al., 1999; i.e., any of the chloroplast genes with a CGC or CGA codon), and is therefore almost certainly pleiotropic, its strong effect on regulation of CEF is of interest, and illustrates some key bioenergetics points.

### Modifying chloroplast translation leads to accumulation of H_2_O_2_, a likely activator of CEF

It was previously shown that simply suppressing overall photosynthesis does not, by itself, trigger high rates of CEF (Cruz et al., 2005; Livingston et al., 2010b), implying that the effect of *hcef2* on CEF is not caused by a general effect on photosynthetic capacity, but more likely affecting a CEF regulatory process. We do not see major changes in NDH protein levels (Figure 14) suggesting that the *hcef* affects activation of NDH at the enzyme level.

Intriguingly, it has recently been shown that there is a strong relationship between activation of NDH-CEF and the production of H_2_O_2_ in the chloroplast (Casano et al., 2001; Lascano et al., 2003; Livingston et al., 2010b; Takahashi et al., 2013; Strand et al., 2015). Several mutants of the CBB cycle have found to have high CEF (Gotoh et al., 2010; Livingston et al., 2010a,b), one of which was shown to accumulate H_2_O_2_ (Strand et al., 2015), which, within the chloroplast leads to a rapid increase of CEF *in vivo* and accumulation of the complex over longer time periods (Casano et al., 2001; Lascano et al., 2003; Strand et al., 2015). Our observation of a strongly increased H_2_O_2_ production in *hcef2* (Figure 15) supports this model for CEF activation, in agreement with the results from *hcef1* and other high CEF mutants (Livingston et al., 2010b; Strand et al., 2015).

How H_2_O_2_ production leads to the activation of CEF remains an open question, but our results allow us to define some possible mechanisms that have substantial impact on the connections between the status of the cell and the regulation of photosynthesis. It is possible that H_2_O_2_ acts as a direct signaling agent, e.g., by activating a signal cascade ending in phosphorylation of the NDH complex (Lascano et al., 2003). In this case, H_2_O_2_ could be a good indicator of imbalances in the production and consumption of ATP/NADPH (Strand et al., 2015). For instance, a relative deficit of ATP would slow the CBB cycle, leading to accumulation of reduced NADPH and Fd that can reduce O_2_ to superoxide and H_2_O_2_. Activating CEF would correct this imbalance by supplying additional ATP and slowing overall photosynthetic electron transfer by acidifying the thylakoid lumen.

Alternatively, H_2_O_2_ could impact the energy budget of photosynthesis in by leading to the depletion of ATP (discussed below), which in turn could activate CEF. For example, it was proposed that elevated CEF in glyceraldehyde phosphate dehydrogenase-deficient mutations of tobacco is caused by activation of a futile cycle that depletes the chloroplast of ATP (Livingston et al., 2010b). Similarly, it has recently been proposed that loss of chloroplast FBPase activity induces a glucose-6-phosphate shunt, allowing photosynthesis to continue despite the lesion in FBPase (Sharkey and Weise, 2015). This bypass would impose a higher ATP demand, and that this increase in ATP demand could be leading to the upregulation of CEF. It is thus also possible that H_2_O_2_ production in *hcef2* could lead to ATP deficits through induction or similar futile cycling related to disruption of the normal redox-regulation of enzymes.

There are several (non-exclusive) possible mechanisms for the accumulation of high levels of H_2_O_2_ in *hcef2*. Because the codons affected in *hcef2* (and *tada1*) are present in >80% of the protein coding genes within the chloroplast genome (Sato et al., 1999), the overall effect on plastid translation is expected to similar to that of *prsp3*, and *rps17*, but through a different mechanism. In other words, *hcef2, tada1, prsp3*, and *hcef1* all showed both increased levels of H_2_O_2_ (Figure 15) and elevated CEF (Figures 4, 8, 10). This suggests a model in which defects in chloroplast translation lead to discoordination of protein homeostasis, and indirectly to elevated H_2_O_2_, and thus the activation of CEF. Evidence for such discoordination in *hcef2* can be seen in the altered ratios of photosynthetic complexes, cytochrome *bf* and PSI (Figures 12A,C) and in the preferential loss of PSI subunits PSAL and PSAF (Figure 14).

It is possible that this dis-coordination could increase the rates of H_2_O_2_ production or decrease the effectiveness of the chloroplast H_2_O_2_ detoxification system. The majority of H_2_O_2_ production in chloroplasts is proposed to arise from the accumulation of PSI reduced oxygen radicals, through the Mehler reaction, of which H_2_O_2_ is an intermediate step in the detoxification (or water-water cycle; Mubarakshina et al., 2010). In this scenario, the increased H_2_O_2_ production in *hcef2* could be explained by partial protonic uncoupling of the thylakoid membrane as suggested by the ECS decay kinetics (Figure 11), which would decrease the production of ATP relative to NADPH, leading to both ATP deficits and accumulation of electrons on acceptor side of PSI which in turn can lead to reduction of O_2_. In addition to the effects of uncoupling, the loss of PSI peripheral subunits in *hcef2* (Figure 14) may lead a change in the donor/acceptor environment on the stromal side of PSI, altering the rates of electron transfer to soluble carriers, and potentially leading to the increased reduction of the molecular oxygen. This defect is hinted at in a PSAE deficient mutant, when marker gene expression is used as a ROS indicator (Ihnatowicz et al., 2007).

Finally, it is worth noting that we obtained high CEF mutants that have defects in very diverse processes. On one hand, this outcome is frustrating because it makes it less likely that genetic approaches, screening for high CEF mutants, will directly indicate the precise mechanism of CEF activation. On the other hand, it indicates just how integrated the NDH-CEF response is in balancing the overall metabolic system of the organism. In other words, it is able to respond to a wide range of system-wide perturbations related to energy imbalances, and most likely transmitted to the NDH complex through a common energy currency (e.g., ATP) or general signal (e.g., H_2_O_2_).

## Methods

### Plant materials and growth

All plants were grown photoautotrophically on soil in a controlled growth chamber with a 16:8 h light/dark photoperiod (~100 μmols photons m^−2^ s^−1^, white light) at 22°C. Seed for *tada1* (GK-119G08) and the *tada1* line complimented with P35S:ΔNTADA1 was graciously provided by Dr. Jośe Gualberto. Seed for *prsp3* (Salk_010806) and *rps17* (Salk_066943) were provided by the ABRC. The *tada1* insertion was verified with as described in Delannoy et al. (2009). The *hcef2* mutation in the *tada1* locus was verified by sequencing. The presence of the P35S: ΔNTADA1 construct was verified using primers for the 35S promoter (5′-CCACTGACGTAAGGGATGACG-3′) and the C-terminus end of TADA1 (5′-TGCTTTAGAACCCTCTCGAAT-3′). Verification of homozygous *prsp3* and *rps17* was performed using primers generated from the SIGnAL T-DNA primer design tool (http://signal.salk.edu/tdnaprimers.2.html).

### Isolation of *hcef* mutants

The *hcef2* mutant was initially identified and isolated as a high NPQ mutant as described in Livingston et al. (2010a). Identification of backcrossed lines and F2 mapping populations with high NPQ was performed as described in Livingston et al. (2010a).

### 77 K fluorescence spectroscopy

Fresh light adapted leaf material was flash frozen in liquid nitrogen, ground to a fine powder and diluted to <5 μg chlorophyll ml^−1^ in ice as described in Weis (1985). Emission spectra were detected using a spectrofluorometer (Ocean Optics, HR200+ES) by a blue (440 nm) diode laser, controlled by SpectraSuite software (Ocean Optics). The spectra were normalized to the 735 nm peak.

### *In vivo* and *in vitro* spectroscopy

All *in vivo* spectroscopic measurements were performed on fully expanded leaves in mature plants just prior to bolting (Figure 1). Comparisons were made between mature leaves, despite age, due to the inhibited growth in the mutant lines. Steady-state chlorophyll *a* fluorescence yield and light induced absorption changes were made as extensively described elsewhere (Genty et al., 1989; Kanazawa and Kramer, 2002; Avenson et al., 2005; Baker et al., 2007; Baker, 2008; Livingston et al., 2010a,b) on a spectrophotometer/fluorimeter described in Hall et al. (2013). Prior to the experimental protocol, plants were dark adapted for 10 min. Steady state was reached, and measurements were made after 10 min of actinic illumination. To account for changes in pigmentation of the mutants, LEF was calculated using the approach of Dai et al. (1996) and Livingston et al. (2010a) using the following equation:

$$\begin{array}{l} \text{LEF }=\;\Phi_{\text{II}}{\;}^{*}\;i{\;}^{*}\;A{\;}^{*}\;0.5 \end{array}$$

where *i* is the actinic light intensity and *A* is the absorptivity of the leaf quantified as in Livingston et al. (2010a). Leaf absorptivity used for each genotype was the average of three leaves, and calculated for each new batch of plants grown. Absorptivity values in *hcef2* and *tada1* (0.64 and 0.65, respectively) were lower than Col-0 (0.83, *p* < 0.01, *n* = 3), but not significantly different from each other (*p* = 0.99, *n* = 3), while there was no significant difference between Col-0 and the complimented line (0.83, 0.85, respectively, *p* = 0.65, *n* = 3).

Electrochromic shift measurements were corrected for changes in leaf properties by normalizing to leaf chlorophyll content. This correction gives similar results as corrections described in Avenson et al. (2005) and Livingston et al. (2010a,b).

For chlorophyll *a* fluorescence yield in response to short actinic pulses, plants were dark adapted for 10 min prior to the experiment. For each trace the excitation light was pulsed at a frequency of 500 Hz, contributing minimally to the kinetics of chlorophyll *a* fluorescence induction in the absence of actinic illumination. Five actinic flashes (10 ms at ~12,000 μmol photons m^−2^ s^−1^) were given 0.2 s apart. A second experiment was performed in which an interval of far-red illumination was inserted after the last actinic flash. Finally, the experiment was repeated in the dark with an interval of far-red illumination. Data was normalized to F0 and Fm of the initial experiment, and the baseline was set to 0.

For *in vitro* spectroscopy, broken chloroplasts were prepared as described in Fisher and Kramer (2014) at the same developmental stage as described above. Cytochrome α band redox difference spectra of chloroplast preparations were obtained using a Perkin-Elmer-Lambda 650 UV/vis spectrophotometer.

### Map-based cloning of *hcef2*

The *hcef2* mutant was mapped on chromosome 1 between At1G68560 (25,733,701 bp) and At1G69020 (25,947,401), a 213.7 kb region, using molecular markers based on Simple sequence length polymorphisms (SSLPs) and cleaved amplified polymorphic sequences (CAPS; Konieczny and Ausubel, 1993). Polymorphism sequence information between Col-0 and Landsberg *erecta*, from the ABRC TAIR website (http://www.arabidopsis.org/browse/Cereon/index.jsp), was used to design SSLPs and/or CAPS marker for mapping. F2 plants were derived from breeding homozygous *hcef2* (Col-0 background) and wildtype (Landsberg *erecta* background). The *hcef2* mutation was found to be recessive, and genomic DNA was isolated from homozygous F2 plants (*hcef2 hcef2)* with high NPQ by chlorophyll *a* fluorescence imaging (described above). To determine the *hcef2* mutation, we performed whole genome sequencing on homozygous *hcef2* plants. Genomic DNA from the mutant line was used to create a sequencing library using the Illumina TruSeq DNA Library Kit following manufacturers recommendations. The library was then sequenced on an Illumina GAIIx using single end with 50 base reads. All next generation sequencing was conducted by the Genomics Core of the Research Technology Support Facility at Michigan State University. Illumina reads were assembled using SeqMan NGen software (DNASTAR). SNPs were compared across multiple samples of the same Col-0 background and SNPs unique to *hcef2* were confirmed by Sanger sequencing.

### CAPS marker for *hcef2* genotype

To genotype *hcef2* without sequencing a CAPS marker was designed for the *hcef2* mutation. A PCR fragment spanning the mutation site was amplified (5′-GAGGCTGATTGGTCAAGGA-3′, forward; 5′-GGATGTTCAAAGGCTGTGGT-3′, reverse) and then digested with NruI (R0192, New ENGLAND BioLabs). The Col-0 sequence was cut with NruI, and homozygocity/heterozygocity was determined by comparing banding patterns on a 2% agarose gel.

### Infiltrations

Freshly detached leaves were infiltrated with 20 μM antimycin A in distilled water in the dark by soaking between two saturated lab tissues for 3 h. Successful infiltration of antimycin A was confirmed by secondary effects of this chemical on NPQ responses probed by chlorophyll fluorescence (Oxborough and Horton, 1987). Leaves were infiltrated with 100 μM methyl viologen in a similar manner for 1 h prior to measurement.

### H_2_O_2_ quantification

To quantify relative H_2_O_2_ accumulation, leaves were flash frozen in the light and H_2_O_2_ was extracted in 50 mM potassium phosphate (pH 7.4). Total H_2_O_2_ of the extract was quantified using Amplex Red fluorescence (Invitrogen) against a standard curve, and normalized to chlorophyll content of the sample, then normalized to average Col-0 H_2_O_2_ content.

### Determination of protein changes in *hcef2*

Thylakoids were prepared from leaves of *hcef2* and Col-0 as described in Strand et al. (2016). For one dimensional SDS-PAGE, thylakoids were solubilized in Laemmli buffer and loaded on a total chlorophyll basis for simplicity as there were no large differences seen in protein load from the coomassie stain (Figure 14A). For western blots, proteins were transferred to a PVDF membrane and probed with antibodies raised against the whole ATP synthase (Agrisera), cytochrome *b*_6_ (Agrisera), and/or cytochrome *f* (Agrisera). All blots were performed with *n* = 3, with the exception of the ATP synthase blot, which was performed *n* = 4.

## Author contributions

DDS designed the research, performed research, analyzed data, and wrote the paper. AKL and NF designed the research, performed research, analyzed data, and wrote portions of the paper. MS-C, DM, KKH, and JEF designed the research, performed research, and analyzed data. ML and TK analyzed data. HME performed research. JAC designed the research, performed research, contributed new analytic tools, and analyzed data. AD and KK designed the research. DMK designed the research, interpreted the data, and wrote the paper.

## Funding

Experiments performed at MSU and WSU were funded by Grant DE-FG02-11ER16220 from the Division of Chemical Sciences, Geosciences, and Biosciences, Office of Basic Energy Sciences of the US Department of Energy (to DK), with support for the development and use of phenotyping tools from U.S. Department of Energy (DOE), Office of Science, Basic Energy Sciences (BES DE-FG02-91ER20021) and the MSU Center for Advanced Algal and Plant Phenotyping (CAAPP). A portion of this work was performed in the Environmental Molecular Science Laboratory, a U.S. Department of Energy national scientific user facility at Pacific Northwest National Laboratory (PNNL) in Richland, WA.

### Conflict of interest statement

The authors declare that the research was conducted in the absence of any commercial or financial relationships that could be construed as a potential conflict of interest. The handling Editor declared a past co-authorship with one of the authors HE and states that the process nevertheless met the standards of a fair and objective review.

## References

[B1] Alric J. (2014). Redox and ATP control of photosynthetic cyclic electron flow in *Chlamydomonas reinhardtii*: (II) involvement of the PGR5-PGRL1 pathway under anaerobic conditions. Biochim. Biophys. Acta 1837, 825–834. 10.1016/j.bbabio.2014.01.024 24508216

[B2] Avenson T. J. Cruz J. A. Kanazawa A. Kramer D. M. (2005). Regulating the proton budget of higher plant photosynthesis. Proc. Natl. Acad. Sci. U.S.A. 102, 9709–9713. 10.1073/pnas.0503952102 15972806 PMC1172270

[B3] Avenson T. J. Cruz J. A. Kramer D. M. (2004). Modulation of energy-dependent quenching of excitons in antennae of higher plants. Proc. Natl. Acad. Sci. U.S.A. 101, 5530–5535. 10.1073/pnas.0401269101 15064404 PMC397417

[B4] Baker N. R. (2008). Chlorophyll fluorescence: a probe of photosynthesis *in vivo*. Annu. Rev. Plant Biol. 59, 89–113. 10.1146/annurev.arplant.59.032607.092759 18444897

[B5] Baker N. R. Harbinson J. Kramer D. M. (2007). Determining the limitations and regulation of photosynthetic energy transduction in leaves. Plant Cell Environ. 30, 1107–1125. 10.1111/j.1365-3040.2007.01680.x 17661750

[B6] Bendall D. S. Manasse R. S. (1995). Cyclic photophosphorylation and electron transport. Biochim. Biophys. Acta 1229, 23–38. 10.1016/0005-2728(94)00195-B

[B7] Breyton C. Nandha B. Johnson G. N. Joliot P. Finazzi G. (2006). Redox modulation of cyclic electron flow around photosystem I in C3 plants. Biochemistry 45, 13465–13475. 10.1021/bi061439s 17087500

[B8] Burrows P. A. Sazanov L. A. Svab Z. Maliga P. Nixon P. J. (1998). Identification of a functional respiratory complex in chloroplasts through analysis of tobacco mutants containing disrupted plastid ndh genes. EMBO J. 17, 868–876. 10.1093/emboj/17.4.868 9463365 PMC1170436

[B9] Cape J. L. Bowman M. K. Kramer D. M. (2006). Understanding the cytochrome bc complexes by what they don't do. The Q-cycle at 30. Trends Plant Sci. 11, 46–55. 10.1016/j.tplants.2005.11.007 16352458

[B10] Casano L. M. Martín M. Sabater B. (2001). Hydrogen peroxide mediates the induction of chloroplastic Ndh complex under photooxidative stress in barley. Plant Physiol. 125, 1450–1458. 10.1104/pp.125.3.1450 11244124 PMC65623

[B11] Cleland R. E. Bendall D. S. (1992). Photosystem I cyclic electron transport: measurement of ferredoxin-plastoquinone reductase activity. Photosyn. Res. 34, 409–418. 10.1007/BF00029815 24408836

[B12] Cramer W. A. Hasan S. S. Yamashita E. (2011). The Q cycle of cytochrome bc complexes: a structure perspective. Biochim. Biophys. Acta 1807, 788–802. 10.1016/j.bbabio.2011.02.006 21352799 PMC3101715

[B13] Cruz J. A. Avenson T. J. Kanazawa A. Takizawa K. Edwards G. E. Kramer D. M. (2005). Plasticity in light reactions of photosynthesis for energy production and photoprotection. J. Exp. Bot. 56, 395–406. 10.1093/jxb/eri022 15533877

[B14] Dai Z. Ku M. B. Edwards G. (1996). Oxygen sensitivity of photosynthesis and photorespiration in different photosynthetic types in the genus Flaveria. Planta 198, 563–571. 10.1007/BF00262643 28321667

[B15] DalCorso G. Pesaresi P. Masiero S. Aseeva E. Schünemann D. Finazzi G.. (2008). A complex containing PGRL1 and PGR5 is involved in the switch between linear and cyclic electron flow in Arabidopsis. Cell 132, 273–285. 10.1016/j.cell.2007.12.028 18243102

[B16] Delannoy E. Le Ret M. Faivre-Nitschke E. Estavillo G. M. Bergdoll M. Taylor N. L.. (2009). Arabidopsis tRNA adenosine deaminase arginine edits the wobble nucleotide of chloroplast tRNAArg(ACG) and is essential for efficient chloroplast translation. Plant Cell 21, 2058–2071. 10.1105/tpc.109.066654 19602623 PMC2729595

[B17] Eberhard S. Finazzi G. Wollman F.-A. (2008). The dynamics of photosynthesis. Annu. Rev. Genet. 42, 463–515. 10.1146/annurev.genet.42.110807.091452 18983262

[B18] Fan D.-Y. Nie Q. Hope A. B. Hillier W. Pogson B. J. Chow W. S. (2007). Quantification of cyclic electron flow around Photosystem I in spinach leaves during photosynthetic induction. Photosyn. Res. 94, 347–357. 10.1007/s11120-006-9127-z 17211579

[B19] Finazzi G. Rappaport F. Furia A. Fleischmann M. Rochaix J.-D. Zito F.. (2002). Involvement of state transitions in the switch between linear and cyclic electron flow in *Chlamydomonas reinhardtii*. EMBO Rep. 3, 280–285. 10.1093/embo-reports/kvf047 11850400 PMC1084013

[B20] Fisher N. Kramer D. M. (2014). Non-photochemical reduction of thylakoid photosynthetic redox carriers *in vitro*: relevance to cyclic electron flow around photosystem I? Biochim. Biophys. Acta 1837, 1944–1954. 10.1016/j.bbabio.2014.09.005 25251244

[B21] Genty B. Briantais J.-M. Baker N. R. (1989). The relationship between the quantum yield of photosynthetic electron transport and quenching of chlorophyll fluorescence. Biochim. Biophys. Acta 990, 87–92. 10.1016/S0304-4165(89)80016-9

[B22] Gotoh E. Matsumoto M. Ogawa K. Kobayashi Y. Tsuyama M. (2010). A qualitative analysis of the regulation of cyclic electron flow around photosystem I from the post-illumination chlorophyll fluorescence transient in Arabidopsis: a new platform for the *in vivo* investigation of the chloroplast redox state. Photosyn. Res. 103, 111–123. 10.1007/s11120-009-9525-0 20054711

[B23] Hall C. C. Cruz J. Wood M. Zegarac R. DeMars D. Carpenter J. (2013). Photosynthetic measurements with the Idea Spec: an integrated diode emitter array spectrophotometer/fluorometer, in Photosynthesis Research for Food, Fuel and Future, eds Kuang T. Lu C. Zhang L. (Hangzhou; Heidelberg: Zhejiang University Press; Springer), 184–188

[B24] Havaux M. Rumeau D. Ducruet J.-M. (2005). Probing the FQR and NDH activities involved in cyclic electron transport around Photosystem I by the “afterglow” luminescence. Biochim. Biophys. Acta 1709, 203–213. 10.1016/j.bbabio.2005.07.010 16137641

[B25] Hope A. B. Valente P. Matthews D. B. (1994). Effects of pH on the kinetics of redox reactions in and around the cytochromebf complex in an isolated system. Photosyn. Res. 42, 111–120. 10.1007/BF02187122 24306499

[B26] Huang W. Yang S.-J. Zhang S.-B. Zhang J.-L. Cao K.-F. (2012). Cyclic electron flow plays an important role in photoprotection for the resurrection plant *Paraboea rufescens* under drought stress. Planta 235, 819–828. 10.1007/s00425-011-1544-3 22080919

[B27] Huang W. Zhang S.-B. Cao K.-F. (2010). Stimulation of cyclic electron flow during recovery after chilling-induced photoinhibition of PSII. Plant Cell Physiol. 51, 1922–1928. 10.1093/pcp/pcq144 20861006

[B28] Ihnatowicz A. Pesaresi P. Leister D. (2007). The E subunit of photosystem I is not essential for linear electron flow and photoautotrophic growth in *Arabidopsis thaliana*. Planta 226, 889–895. 10.1007/s00425-007-0534-y 17503073

[B29] Iwai M. Takizawa K. Tokutsu R. Okamuro A. Takahashi Y. Minagawa J. (2010). Isolation of the elusive supercomplex that drives cyclic electron flow in photosynthesis. Nature 464, 1210–1213. 10.1038/nature08885 20364124

[B30] Johnson X. Steinbeck J. Dent R. M. Takahashi H. Richaud P. Ozawa S.-I.. (2014). Proton gradient regulation 5-mediated cyclic electron flow under ATP- or redox-limited conditions: a study of ΔATPase pgr5 and ΔrbcL pgr5 mutants in the green alga *Chlamydomonas reinhardtii*. Plant Physiol. 165, 438–452. 10.1104/pp.113.233593 24623849 PMC4012601

[B31] Joliot P. Joliot A. (2002). Cyclic electron transfer in plant leaf. Proc. Natl. Acad. Sci. U.S.A. 99, 10209–10214. 10.1073/pnas.102306999 12119384 PMC126649

[B32] Joliot P. Joliot A. (2006). Cyclic electron flow in C3 plants. Biochim. Biophys. Acta 1757, 362–368. 10.1016/j.bbabio.2006.02.018 16762315

[B33] Kanazawa A. Kramer D. M. (2002). *In vivo* modulation of nonphotochemical exciton quenching (NPQ) by regulation of the chloroplast ATP synthase. Proc. Natl. Acad. Sci. U.S.A. 99, 12789–12794. 10.1073/pnas.182427499 12192092 PMC130538

[B34] Karcher D. Bock R. (2009). Identification of the chloroplast adenosine-to-inosine tRNA editing enzyme. RNA 15, 1251–1257. 10.1261/rna.1600609 19460869 PMC2704073

[B35] Klughammer C. Schreiber U. (1994). An improved method, using saturating light pulses, for the determination of photosystem I quantum yield via P700^+^-absorbance changes at 830 nm. Planta 192, 261–268. 10.1007/BF01089043

[B36] Kohzuma K. Cruz J. A. Akashi K. Hoshiyasu S. Munekage Y. N. Yokota A.. (2009). The long-term responses of the photosynthetic proton circuit to drought. Plant Cell Environ. 32, 209–219. 10.1111/j.1365-3040.2008.01912.x 19021886

[B37] Konieczny A. Ausubel F. M. (1993). A procedure for mapping Arabidopsis mutations using co-dominant ecotype-specific PCR-based markers. Plant J. 4, 403–410. 10.1046/j.1365-313X.1993.04020403.x 8106085

[B38] Kramer D. M. Avenson T. J. Edwards G. E. (2004). Dynamic flexibility in the light reactions of photosynthesis governed by both electron and proton transfer reactions. Trends Plant Sci. 9, 349–357. 10.1016/j.tplants.2004.05.001 15231280

[B39] Kramer D. M. Crofts A. R. (1989). Activation of the chloroplast ATPase measured by the electrochromic change in leaves of intact plants. Biochim. Biophys. Acta 976, 28–41. 10.1016/S0005-2728(89)80186-0

[B40] Kramer D. M. Crofts A. R. (1994). Re-examination of the properties and function of the b cytochromes of the thylakoid cytochrome bf complex. Biochim. Biophys. Acta 1184, 193–201. 10.1016/0005-2728(94)90223-2

[B41] Kramer D. M. Evans J. R. (2011). The importance of energy balance in improving photosynthetic productivity. Plant Physiol. 155, 70–78. 10.1104/pp.110.166652 21078862 PMC3075755

[B42] Krause G. H. Weis E. (1984). Chlorophyll fluorescence as a tool in plant physiology : II. Interpretation of fluorescence signals. Photosynth. Res. 5, 139–157. 10.1007/BF00028527 24458602

[B43] Lascano H. R. Casano L. M. Martín M. Sabater B. (2003). The activity of the chloroplastic Ndh complex is regulated by phosphorylation of the NDH-F subunit. Plant Physiol. 132, 256–262. 10.1104/pp.103.020321 12746530 PMC166970

[B44] Li X.-P. Muller-Moule P. Gilmore A. M. Niyogi K. K. (2002). PsbS-dependent enhancement of feedback de-excitation protects photosystem II from photoinhibition. Proc. Natl. Acad. Sci. U.S.A. 99, 15222–15227. 10.1073/pnas.232447699 12417767 PMC137571

[B45] Livingston A. K. Cruz J. A. Kohzuma K. Dhingra A. Kramer D. M. (2010a). An Arabidopsis mutant with high cyclic electron flow around photosystem I (hcef) involving the NADPH dehydrogenase complex. Plant Cell 22, 221–233. 10.1105/tpc.109.071084 20081115 PMC2828696

[B46] Livingston A. K. Kanazawa A. Cruz J. A. Kramer D. M. (2010b). Regulation of cyclic electron flow in C(3) plants: differential effects of limiting photosynthesis at ribulose-1,5-bisphosphate carboxylase/oxygenase and glyceraldehyde-3-phosphate dehydrogenase. *Plant*. Cell Environ. 33, 1779–1788. 10.1111/j.1365-3040.2010.02183.x 20545877

[B47] Lucker B. Kramer D. M. (2013). Regulation of cyclic electron flow in *Chlamydomonas reinhardtii* under fluctuating carbon availability. Photosyn. Res. 117, 449–459. 10.1007/s11120-013-9932-0 24113925

[B48] Mubarakshina M. M. Ivanov B. N. Naydov I. A. Hillier W. Badger M. R. Krieger-Liszkay A. (2010). Production and diffusion of chloroplastic H2O2 and its implication to signalling. J. Exp. Bot. 61, 3577–3587. 10.1093/jxb/erq171 20595239

[B49] Müller P. Li X. P. Niyogi K. K. (2001). Non-photochemical quenching. A response to excess light energy. Plant Physiol. 125, 1558–1566. 10.1104/pp.125.4.1558 11299337 PMC1539381

[B50] Munekage Y. Hashimoto M. Miyake C. (2004). Cyclic electron flow around photosystem I is essential for photosynthesis. Nature 700, 0–3. 10.1038/nature02598 15175756

[B51] Munekage Y. Hojo M. Meurer J. Endo T. Tasaka M. Shikanai T. (2002). PGR5 is involved in cyclic electron flow around photosystem I and is essential for photoprotection in Arabidopsis. Cell 110, 361–371. 10.1016/S0092-8674(02)00867-X 12176323

[B52] Oxborough K. Horton P. (1987). Characterisation of the effects of Antimycin A upon high energy state quenching of chlorophyll fluorescence (qE) in spinach and pea chloroplasts. Photosynth. Res. 12, 119–127. 10.1007/bf00047942 24435635

[B53] Sacksteder C. A. Kramer D. M. (2000). Dark-interval relaxation kinetics (DIRK) of absorbance changes as a quantitative probe of steady-state electron transfer. Photosyn. Res. 66, 145–158. 10.1023/A:1010785912271 16228416

[B54] Sato S. Nakamura Y. Kaneko T. Asamizu E. Tabata S. (1999). Complete structure of the chloroplast genome of *Arabidopsis thaliana*. DNA Res. 6, 283–290. 10.1093/dnares/6.5.283 10574454

[B55] Sazanov L. A. Burrows P. A. Nixon P. J. (1998). The plastid ndh genes code for an NADH-specific dehydrogenase: isolation of a complex I analogue from pea thylakoid membranes. Proc. Natl. Acad. Sci. U.S.A. 95, 1319–1324. 10.1073/pnas.95.3.1319 9448329 PMC18756

[B56] Sharkey T. D. Weise S. E. (2015). The glucose 6-phosphate shunt around the Calvin – Benson cycle. J. Exp. Bot. 67, 4067–4077. 10.1093/jxb/erv484 26585224

[B57] Shikanai T. Endo T. Hashimoto T. Yamada Y. Asada K. Yokota A. (1998). Directed disruption of the tobacco ndhB gene impairs cyclic electron flow around photosystem I. Proc. Natl. Acad. Sci. U.S.A. 95, 9705–9709. 10.1073/pnas.95.16.9705 9689145 PMC21403

[B58] Strand D. D. Fisher N. Davis G. A. Kramer D. M. (2016). Redox regulation of the antimycin A sensitive pathway of cyclic electron flow around photosystem I in higher plant thylakoids. Biochim. Biophys. Acta 1857, 1–6. 10.1016/j.bbabio.2015.07.012 26235611

[B59] Strand D. D. Kramer D. M. (2014). Control of non-photochemical exciton quenching by the proton circuit of photosynthesis, in Advances in Photosynthesis and Respiration, Vol. 40, Non-Photochemical Quenching and Energy Dissipation in Plants, Algae and Cyanobacteria Demmig-Adams, eds Garab B. Adams G. III Govindjee W. (Dordrecht: Springer Netherlands), 387–408.

[B60] Strand D. D. Livingston A. K. Satoh-cruz M. Froehlich J. E. Maurino V. G. Kramer D. M. (2015). Activation of cyclic electron flow by hydrogen peroxide *in vivo*. Proc. Natl. Acad. Sci. U.S.A. 112, 5539–5544. 10.1073/pnas.1418223112 25870290 PMC4418880

[B61] Tagawa K. Tsujimoto H. Y. arnon D. I. (1963). Separation by monochromatic light of photosynthetic phosphorylation from oxygen evolution. Proc. Natl. Acad. Sci. U.S.A. 50, 544–549. 10.1073/pnas.50.3.544 14067103 PMC221216

[B62] Takabayashi A. Kishine M. Asada K. Endo T. Sato F. (2005). Differential use of two cyclic electron flows around photosystem I for driving CO2-concentration mechanism in C4 photosynthesis. Proc. Natl. Acad. Sci. U.S.A. 102, 16898–16903. 10.1073/pnas.0507095102 16272223 PMC1283823

[B63] Takahashi H. Clowez S. Wollman F.-A. Vallon O. Rappaport F. (2013). Cyclic electron flow is redox-controlled but independent of state transition. Nat. Commun. 4, 1954. 10.1038/ncomms2954 23760547 PMC3709502

[B64] Takahashi S. Milward S. E. Fan D.-Y. Chow W. S. Badger M. R. (2009). How does cyclic electron flow alleviate photoinhibition in Arabidopsis? Plant Physiol. 149, 1560–1567. 10.1104/pp.108.134122 19118124 PMC2649389

[B65] Takizawa K. Cruz J. A. Kanazawa A. Kramer D. M. (2007). The thylakoid proton motive force *in vivo*. Quantitative, non-invasive probes, energetics, and regulatory consequences of light-induced pmf. Biochim. Biophys. Acta 1767, 1233–1244. 10.1016/j.bbabio.2007.07.006 17765199

[B66] Terashima M. Petroutsos D. Hüdig M. Tolstygina I. Trompelt K. Gäbelein P.. (2012). Calcium-dependent regulation of cyclic photosynthetic electron transfer by a CAS, ANR1, and PGRL1 complex. Proc. Natl. Acad. Sci. U.S.A. 109, 17717–17722. 10.1073/pnas.1207118109 23045639 PMC3491457

[B67] Tiller N. Weingartner M. Thiele W. Maximova E. Schöttler M. A. Bock R. (2012). The plastid-specific ribosomal proteins of *Arabidopsis thaliana* can be divided into non-essential proteins and genuine ribosomal proteins. Plant J. 69, 302–316. 10.1111/j.1365-313X.2011.04791.x 21923745

[B68] Weis E. (1985). Chlorophyll fluorescence at 77K in intact leaves: characterization of a technique to eliminate artifacts related to self-absorption. Photosyn. Res. 6, 73–86. 10.1007/BF00029047 24442829

[B69] Zhang H. Whitelegge J. P. Cramer W. A. (2001). Ferredoxin:NADP+ oxidoreductase is a subunit of the chloroplast cytochrome b6f complex. J. Biol. Chem. 276, 38159–38165. 10.1074/jbc.M105454200 11483610

